# Interactions of Cultures and Top People of Wikipedia from Ranking of 24 Language Editions

**DOI:** 10.1371/journal.pone.0114825

**Published:** 2015-03-04

**Authors:** Young-Ho Eom, Pablo Aragón, David Laniado, Andreas Kaltenbrunner, Sebastiano Vigna, Dima L. Shepelyansky

**Affiliations:** 1 Laboratoire de Physique Théorique du CNRS, IRSAMC, Université de Toulouse, UPS, F-31062 Toulouse, France; 2 Barcelona Media Foundation, Barcelona, Spain; 3 Dipartimento di Informatica, Università degli Studi di Milano, Milano, Italy; Tianjin University, CHINA

## Abstract

Wikipedia is a huge global repository of human knowledge that can be leveraged to investigate interwinements between cultures. With this aim, we apply methods of Markov chains and Google matrix for the analysis of the hyperlink networks of 24 Wikipedia language editions, and rank all their articles by PageRank, 2DRank and CheiRank algorithms. Using automatic extraction of people names, we obtain the top 100 historical figures, for each edition and for each algorithm. We investigate their spatial, temporal, and gender distributions in dependence of their cultural origins. Our study demonstrates not only the existence of skewness with local figures, mainly recognized only in their own cultures, but also the existence of global historical figures appearing in a large number of editions. By determining the birth time and place of these persons, we perform an analysis of the evolution of such figures through 35 centuries of human history for each language, thus recovering interactions and entanglement of cultures over time. We also obtain the distributions of historical figures over world countries, highlighting geographical aspects of cross-cultural links. Considering historical figures who appear in multiple editions as interactions between cultures, we construct a network of cultures and identify the most influential cultures according to this network.

## Introduction

The influence of digital media on collective opinions, social relationships, and information dynamics is growing significantly with the advances of information technology. On the other hand, understanding how collective opinions are reflected in digital media has crucial importance. Among such a medium, Wikipedia, the open, free, and online encyclopedia, has crucial importance since it is not only the largest global knowledge repository but also the biggest collaborative knowledge platform on the Web. Thanks to its huge size, broad coverage and ease of use, Wikipedia is currently one of the most widely used knowledge references. However, since its beginning, there have been constant concerns about the reliability of Wikipedia because of its openness. Although professional scholars may not be affected by a possible skewness or bias of Wikipedia, students and the public can be affected significantly [1, 2]. Extensive studies have examined the reliability of contents [1–3], topic coverage [4], vandalism [5], and conflict [6–8] in Wikipedia.

Wikipedia is available in different language editions; 287 language editions are currently active. This indicates that the same topic can be described in hundreds of articles written by different language user groups. Since language is one of the primary elements of culture [9], collective cultural biases may be reflected on the contents and organization of each Wikipedia edition. Although Wikipedia adopts a “neutral point of view” policy for the description of contents, aiming to provide unbiased information to the public [10], it is natural that each language edition presents reality from a different angle. To investigate differences and relationships among different language editions, we develop mathematical and statistical methods which treat the huge amount of information in Wikipedia, excluding cultural preferences of the investigators.

Cultural bias or differences across Wikipedia editions have been investigated in previous research [11–17]. A special emphasis was devoted to persons described in Wikipedia articles [12] and their ranking [18, 19]. Indeed, human knowledge, as well as Wikipedia itself, was created by people who are the main actors of its development. Thus it is rather natural to analyze a ranking of people according to the Wikipedia hyper-link network of citations between articles (see network data description below). A cross-cultural study of biographical articles was presented in [20], by building a network of interlinked biographies. Another approach was proposed recently in [21]: the difference in importance of historical figures across Wikipedia language editions is assessed on the basis of the global ranking of Wikipedia articles about persons. This study, motivated by the question “Is an important person in a given culture also important in other cultures?”, showed that there are strong entanglements and local biases of historical figures in Wikipedia. Indeed, the results of the study show that each Wikipedia edition favors persons belonging to the same culture (language), but also that there are cross-Wikipedia top ranked persons, who can be signs of entanglement between cultures. These cross-language historical figures can be used to generate inter-culture networks demonstrating interactions between cultures [21]. Such an approach provides us novel insights on cross-cultural differences across Wikipedia editions. However, in [21] only 9 Wikipedia editions, mainly languages spoken in European, have been considered. Thus a broader set of language editions is needed to offer a more complete view on a global scale.

We note that the analysis of persons’ importance via Wikipedia becomes more and more popular. This is well visible from the appearance of new recent studies for the English Wikipedia [22] and for multiple languages [23]. The analysis of coverage of researchers and academics via Wikipedia is reported in [24].

Here we investigate interactions and skewness of cultures with a broader perspective, using global ranking of articles about persons in 24 Wikipedia language editions. According to Wikipedia [25] these 24 languages cover 59 percent of world population. Moreover, according to Wikipedia [26], our selection of 24 language editions covers the 68 percent of the total number of 30.9 millions of Wikipedia articles in all 287 languages. These 24 editions also cover languages which played an important role in human history including Western, Asian and Arabic cultures.

On the basis of this data set we analyze spatial, temporal, and gender skewness in Wikipedia by analyzing birth place, birth date, and gender of the top ranked historical figures in Wikipedia. We identified overall Western, modern, and male skewness of important historical figures across Wikipedia editions, a tendency towards local preference (i.e. each Wikipedia edition favors historical figures born in countries speaking that edition’s language), and the existence of global historical figures who are highly ranked in most of Wikipedia editions. We also constructed networks of cultures based on cross-cultural historical figures to represent interactions between cultures according to Wikipedia.

To obtain a unified ranking of historical figures for all 24 Wikipedia editions, we introduce an average ranking which gives us the top 100 persons of human history. To assess the alignment of our ranking with previous work by historians, we compare it with the Hart’s list of the top 100 people who, according to him, most influenced human history [27]. We note that Hart “ranked these 100 persons in order of importance: that is, according to the total amount of influence that each of them had on human history and on the everyday lives of other human beings”.

## Methods

In this research, we consider each Wikipedia edition as a network of articles. Each article corresponds to a node of the network and hyperlinks between articles correspond to links of the network. For a given network, we can define an adjacency matrix *A*_*ij*_. If there is a link (one or more) from node (article) *j* to node (article) *i* then *A*_*ij*_ = 1, otherwise, *A*_*ij*_ = 0. The out-degree *k*_*out*_(*j*) is the number of links from node *j* to other nodes and the in-degree *k*_*in*_(*j*) is the number of links to node *j* from other nodes. The links between articles are considered only inside a given Wikipedia edition, there are no links counted between editions. Thus each language edition is analyzed independently from others by the Google matrix methods described below. The transcriptions of names from English to the other 23 selected languages are harvested from WikiData (http://dumps.wikimedia.org/wikidatawiki) and not directly from the text of articles.

To rank the articles of a Wikipedia edition, we use two ranking algorithms based on the articles network structure. Detailed descriptions of these algorithms and their use for Wikipedia editions are given in [18, 19, 28, 29]. The methods used here are described in [21]; we keep the same notations.

### Google matrix

First we construct the matrix *S*_*ij*_ of Markov transitions by normalizing the sum of the elements in each column of *A* to unity (*S*_*ij*_ = *A*_*ij*_/∑_*i*_ *A*_*ij*_, ∑_*i*_ *S*_*ij*_ = 1) and replacing columns with zero elements by elements 1/*N* with *N* being the matrix size. Then the Google matrix is given by the relation *G*_*ij*_ = *αS*_*ij*_ + (1 − *α*)/*N*, where *α* is the damping factor [30]. As in [21] we use the conventional value *α* = 0.85. It is known that the variation of *α* in a range 0.5 ≤ *α* < 0.95 does not significantly affect the probability distribution of ranks discussed below (see e.g. [18, 19, 30]).

### PageRank algorithm

PageRank is a widely used algorithm to rank nodes in a directed network. It was originally introduced for Google web search engine to rank web pages of the World Wide Web based on the idea of academic citations [31]. Currently PageRank is used to rank nodes of network systems from scientific papers [32] to social network services [33], world trade [34] and biological systems [35]. Here we briefly outline the iteration method of PageRank computation. The PageRank vector *P*(*i*, *t*) of a node *i* at iteration *t* in a network with *N* nodes is given by

$$\begin{array}{c} P\left(i,t\right)=\sum_{j}G_{ij}P\left(j,t-1\right)=\left(1-\alpha \right)/N+\alpha \sum_{j}A_{ij}P\left(j,t-1\right)/k_{out}\left(j\right)\;. \end{array}$$ (1)

The stationary state *P*(*i*) of *P*(*i*, *t*) is the PageRank of node *i*. More detailed information about the PageRank algorithm is described in [30]. Ordering all nodes by their decreasing probability *P*(*i*), we obtain the PageRank ranking index *K*(*i*). In qualitative terms, the PageRank probability of a node is proportional to the number of incoming links weighted according to their own probability. A random network surfer spends on a given node a time given on average by the PageRank probability.

### CheiRank algorithm

In a directed network, outgoing links can be as important as ingoing links. In this sense, as a complementary to PageRank, the CheiRank algorithm is defined and used in [18, 28, 36]. The CheiRank vector P*(*i*, *t*) of a node at iteration time *t* is given by

$$\begin{array}{c} P^{*}\left(i\right)=\left(1-\alpha \right)/N+\alpha \underset{j}{\Sigma }A_{ji}P^{*}\left(j\right)/k_{in}\left(j\right) \end{array}$$ (2)

Same as the case of PageRank, we consider the stationary state P*(*i*) of P*(*i*, *t*) as the CheiRank probability of node *i* with *α* = 0.85. High CheiRank nodes in the network have large out-degree. Ordering all nodes by their decreasing probability P*(*i*), we obtain the CheiRank ranking index K*(*i*). The PageRank probability of an article is proportional to the number of incoming links, while the CheiRank probability of an article is proportional to the number of outgoing links. Thus a top PageRank article is important since other articles refer to it, while a top CheiRank article is highly connected because it refers to other articles.

### 2DRank algorithm

PageRank and CheiRank algorithms focus only on in-degree and out-degree of nodes, respectively. The 2DRank algorithm considers both types of information simultaneously to rank nodes with a balanced point of view in a directed network. Briefly speaking, nodes with both high PageRank and CheiRank get high 2DRank ranking. Consider a node *i* which is *K*_*i*_-th ranked by PageRank and K*_i_ ranked by CheiRank. Then we can assign a secondary ranking $K_{i}^{\prime }=max\{K_{i},{K^{*}}_{i}\}$ to the node. If $K_{i}^{\prime }<K_{j}^{\prime }$, then node *j* has lower 2DRank and vice versa. A detailed illustration and description of this algorithm is given in [18].

We note that the studies reported in [21] show that the overlap between top CheiRank persons of different editions is rather small and due to that the statistical accuracy of this data is not sufficient for determining interactions between different cultures for the CheiRank list. Moreover, CheiRank, based on outgoing links only, selects mainly persons from such activity fields like sports and arts where the historical trace is not so important. Due to these reasons we restrict our study to PageRank and 2DRank. It can be also interesting to use other algorithms of ranking, e.g. LeaderRank [37], but here we restrict ourselves to the methods which we already tested, leaving investigation of other raking methods for further studies.

## Data preparation

We consider 24 different language editions of Wikipedia: English (EN), Dutch (NL), German (DE), French (FR), Spanish (ES), Italian (IT), Portuguese (PT), Greek (EL), Danish (DA), Swedish (SV), Polish (PL), Hungarian (HU), Russian (RU), Hebrew (HE), Turkish (TR), Arabic (AR), Persian (FA), Hindi (HI), Malaysian (MS), Thai (TH), Vietnamese (VI), Chinese (ZH), Korean (KO), and Japanese (JA). The Wikipedia data were collected in middle February 2013. The overview summary of each Wikipedia is represented in Table 1.

**Table 1:** Wikipedia hyperlink networks from the 24 considered language editions. Here *N*_*a*_ is the number of articles. Wikipedia data were collected in middle February 2013.

**Edition**	**Language**	***N*_*a*_**	**Edition**	**Language**	***N*_*a*_**
EN	English	4212493	RU	Russian	966284
NL	Dutch	1144615	HE	Hebrew	144959
DE	German	1532978	TR	Turkish	206311
FR	French	1352825	AR	Arabic	203328
ES	Spanish	974025	FA	Persian	295696
IT	Italian	1017953	HI	Hindi	96869
PT	Portuguese	758227	MS	Malaysian	180886
EL	Greek	82563	TH	Thai	78953
DA	Danish	175228	VI	Vietnamese	594089
SV	Swedish	780872	ZH	Chinese	663485
PL	Polish	949153	KO	Korean	231959
HU	Hungarian	235212	JA	Japanese	852087

We understand that our selection of Wikipedia editions does not represent a complete view of all the 287 languages of Wikipedia editions. However, this selection covers most of the largest language editions and allows us to perform quantitative and statistical analysis of important historical figures. Among the 20 largest editions (counted by their size, taken at the middle of 2014) we have not considered the following editions: Waray-Waray, Cebuano, Ukrainian, Catalan, Bokmal-Riksmal, and Finish.

First we ranked all the articles in a given Wikipedia edition by PageRank and 2DRank algorithms, and selected biographical articles about historical figures. To identify biographical articles, we considered all articles belonging to “Category:living people”, or to “Category:Deaths by year” or “Category:Birth by year” or their subcategories in the English Wikipedia. In this way, we obtained a list of about 1.1 million biographical articles. We identified birth place, birth date, and gender of each selected historical figure based on DBpedia [38] or a manual inspection of the corresponding Wikipedia biographical article, when for the considered historical figure no DBpedia data were available. We then started from the list of persons with their biographical article’s title on the English Wikipedia, and found the corresponding titles in other language editions using the inter-language links provided by WikiData. Using the corresponding articles, identified by the inter-languages links in different language editions, we extracted the top 100 persons from the rankings of all Wikipedia articles of each edition. At the end, for each Wikipedia edition and for each ranking algorithm, we have information about the top 100 historical figures with their corresponding name in the English Wikipedia, their birth place and date, and their gender. All 48 lists of the top 100 historical figures in PageRank and 2DRank for the 24 Wikipedia editions and for the two ranking algorithms are represented in [39] and Supporting Information (SI). The original network data for each edition are available at [39]. The automatic extraction of persons from PageRank and 2DRank listings of articles of each edition is performed by using the above whole list of person names in all 24 editions. This method implies a significantly higher recall compared to the manual selection of persons from the ranking list of articles for each edition used in [21].

We attribute each of the 100 historical figures to a birth place at the country level (actual country borders), to a birth date in year, to a gender, and to a cultural group. Historical figures are assigned to the countries currently at the locations where they were born. The cultural group of historical figures is assigned by the most spoken language of their birth place at the current country level. For example, if someone was born in “Constantinople” in the ancient Roman era, since the place is now Istanbul, Turkey, we assign her/his birth place as “Turkey” and since Turkish is the most spoken language in Turkey, we assign this person to the Turkish cultural group. If the birth country does not belong to any of the 24 cultures (languages) which we consider, we assign WR (world) as the culture of this person. We would like to point out that although a culture can not be defined only by language, we think that language is a suitable first-approximation of culture. All lists of top 100 historical figures with their birth place, birth date, gender, and cultural group for each Wikipedia edition and for each ranking algorithm are represented in [39]. A part of this information is also reported in SI.

To apply PageRank and 2DRank methods, we consider each edition as the network of articles of the given edition connected by hyper-links among the articles (see the details of ranking algorithms in Section Methods). The full list of considered Wikipedia language editions is given in Table 1. Table 2 represents the top 10 historical figures by PageRank and 2DRank in the English Wikipedia. Roughly speaking, top PageRank articles imply highly cited articles in Wikipedia and top 2DRank articles imply articles which are both highly cited and highly citing in Wikipedia. In total, we identified 2400 top historical figures for each ranking algorithm. However, since some historical figures such as *Jesus*, *Aristotle*, or *Napoleon* appear in multiple Wikipedia editions, we have 1045 unique top PageRank historical figures and 1616 unique top 2Drank historical figures.

**Table 2:** List of top persons by PageRank and 2DRank for the English Wikipedia. All names are represented by article titles in the English Wikipedia.

**Rank**	**PageRank persons**	**2DRank persons**
1st	Napoleon	Frank Sinatra
2nd	Barack Obama	Michael Jackson
3rd	Carl Linnaeus	Pope Pius XII
4th	Elizabeth II	Elton John
5th	George W. Bush	Elizabeth II
6th	Jesus	Pope John Paul II
7th	Aristotle	Beyoncé Knowles
8th	William Shakespeare	Jorge Luis Borges
9th	Adolf Hitler	Mariah Carey
10th	Franklin D. Roosevelt	Vladimir Putin

We should note that the extraction of persons and their information from a Wikipedia edition is not an easy task even for the English edition, and much more complicated for certain other language editions. Therefore, the above automatic method based on 1.1 million English names and their corresponding names seems to us to be the most adequate approach. Of course, it will miss people who do not have a biographical article on the English Wikipedia. Cross-checking investigation is done for Korean and Russian Wikipedia, which are native languages for two authors, by manually selecting top 100 persons from top lists of all articles ordered by PageRank and 2DRank in both Wikipedia editions. We find that our automatic search misses on average only 2 persons from 100 top persons for these two editions (the missed names are given in SI). The errors appear due to transcription changes of names or missing cases in our name-database based on English Wikipedia. For Western languages the number of errors is presumably reduced since transcription remains close to English. Based on the manual inspection for the Korean and the Russian Wikipedia, we expect that the errors of our automatic recovery of the top people from the whole articles ordered by PageRank and 2DRank are on a level of two percent.

We also note that our study is in compliance with Wikipedia’s Terms and Conditions.

## Results

Above we described the methods used for the extraction of the top 100 persons in the ranking list of each edition. Below we present the obtained results describing the spatial, temporal and gender distributions of top ranked historical figures. We also determine the global and local persons and obtain the network of cultures based on the ranking of persons from a given language by other language editions of Wikipedia.

### Spatial distribution

The birth places of historical figures are attributed to the country containing their geographical location of birth according to the present geographical territories of all world countries. The list of countries appeared for the top 100 persons in all editions is given in Table 3. We also attribute each country to one of the 24 languages of the considered editions. This attribution is done according to the language spoken by the largest part of population in the given country. Thus e.g. Belgium is attributed to Dutch (NL) since the majority of the population speaks Dutch. If the main language of a country is not among our 24 languages, then this country is attributed to an additional section WR corresponding to the remaining world (e.g. Ukraine, Norway are attributed to WR). If the birth place of a person is not known, then it is also attributed to WR. The choice of attribution of a person to a given country in its current geographic territory, and as a result to a certain language, may have some fluctuations due to historical variations of country borders (e.g. Immanuel Kant was born in the current territory of Russia and hence is attributed to Russian language). However, the number of such cases is small, being on a level of 3.5 percent (see Section “Network of cultures” below). We think that the way in which a link between person, language and country is fixed by the birth place avoids much larger ambiguity of attribution of a person according to the native language which is not so easy to fix in an automatic manner.

**Table 3:** List of country code (CC), countries as birth places of historical figures, and language code (LC) for each country. LC is determined by the most spoken language in the given country. Country codes are based on country codes of Internet top-level domains and language codes are based on language edition codes of Wikipedia; WR represents all languages other than the considered 24 languages.

**CC**	**Country**	**LC**	**CC**	**Country**	**LC**	**CC**	**Country**	**LC**
AE	United Arab Emirates	AR	AF	Afghanistan	FA	AL	Albania	WR
AR	Argentina	ES	AT	Austria	DE	AU	Australia	EN
AZ	Azerbaijan	TR	BE	Belgium	NL	BG	Bulgaria	WR
BR	Brazil	PT	BS	Bahamas	EN	BY	Belarus	RU
CA	Canada	EN	CH	Switzerland	DE	CL	Chile	ES
CN	China	ZH	CO	Colombia	ES	CU	Cuba	ES
CY	Cyprus	EL	CZ	Czech Rep.	WR	DE	Germany	DE
DK	Denmark	DA	DZ	Algeria	AR	EG	Egypt	AR
ES	Spain	ES	FI	Finland	WR	FR	France	FR
GE	Georgia	WR	GR	Greece	EL	HK	Hong Kong	ZH
HR	Croatia	WR	HU	Hungary	HU	ID	Indonesia	WR
IE	Ireland	EN	IL	Israel	HE	IN	India	HI
IQ	Iraq	AR	IR	Iran	FA	IS	Iceland	WR
IT	Italy	IT	JP	Japan	JA	KE	Kenya	EN
KG	Kyrgyzstan	WR	KH	Cambodia	WR	KO	S. Korea	KO
KP	N. Korea	KO	KW	Kuwait	AR	KZ	Kazakhstan	WR
LB	Lebanon	AR	LT	Lithuania	WR	LV	Latvia	WR
LY	Libya	AR	MK	Macedonia	WR	MM	Myanmar	WR
MN	Mongolia	WR	MX	Mexico	ES	MY	Malaysia	MS
NL	Netherlands	NL	NO	Norway	WR	NP	Nepal	WR
NZ	New Zealand	EN	OM	Oman	AR	PA	Panama	ES
PE	Peru	ES	PK	Pakistan	HI	PL	Poland	PL
PS	State of Palestine	AR	PT	Portugal	PT	RO	Romania	WR
RS	Serbia	WR	RU	Russia	RU	SA	Saudi Arabia	AR
SD	Sudan	AR	SE	Sweden	SV	SG	Singapore	ZH
SI	Slovenia	WR	SK	Slovakia	WR	SR	Suriname	NL
SY	Syria	AR	TH	Thailand	TH	TJ	Tajikistan	WR
TN	Tunisia	AR	TR	Turkey	TR	TW	Taiwan	ZH
TZ	Tanzania	WR	UA	Ukraine	WR	UK	United Kingdom	EN
US	United States	EN	UZ	Uzbekistan	WR	VE	Venezuela	ES
VN	Vietnam	VI	XX	Unknown	WR	YE	Yemen	AR
ZA	South Africa	WR						

**Fig 1:**
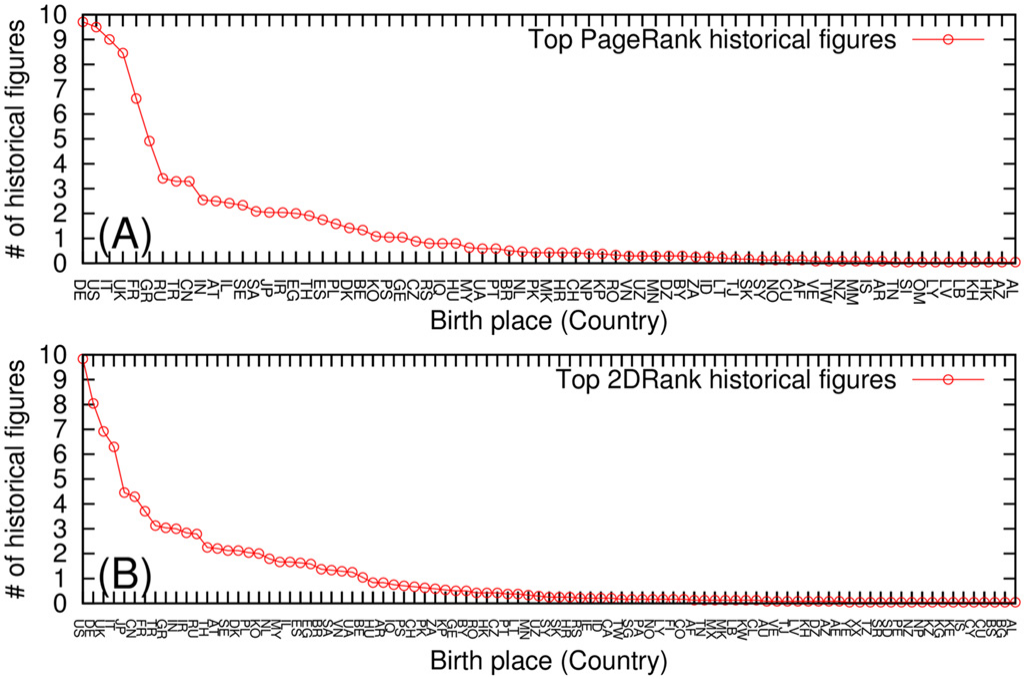
Birth place distribution of top historical figures averaged over 24 Wikipedia edition for (A) PageRank historical figures (71 countries) and (B) 2DRank historical figures (91 countries). Two letter country codes are represented in Table 3.

The obtained spatial distribution of historical figures of Wikipedia over countries is shown in Fig. 1. This averaged distribution gives the average number of top 100 persons born in a specific country as birth place, with averaging done over our 24 Wikipedia editions. Thus an average over the 24 editions gives for Germany (DE) approximately 9.7 persons in the top 100 of PageRank, being at the first position, followed by USA with approximately 9.5 persons. For 2DRank we have USA at the first position with an average of 9.8 persons and Germany at the second with an average of 8.0 persons.

Western (Europe and USA) skewed patterns are observed in both top PageRank historical figures (Fig. 1. (A)) and top 2DRank historical figures (Fig. 1. (B)). This Western skewed pattern is remarkable since 11 Wikipedia editions of the 24 considered editions are not European language editions. Germany, USA, Italy, UK and France are the top five birth places of top PageRank historical figures among 71 countries. On the other hand, USA, Germany, UK, Italy and Japan are top five birth places of the top 2DRank historical figures among 91 countries.

**Fig 2:**
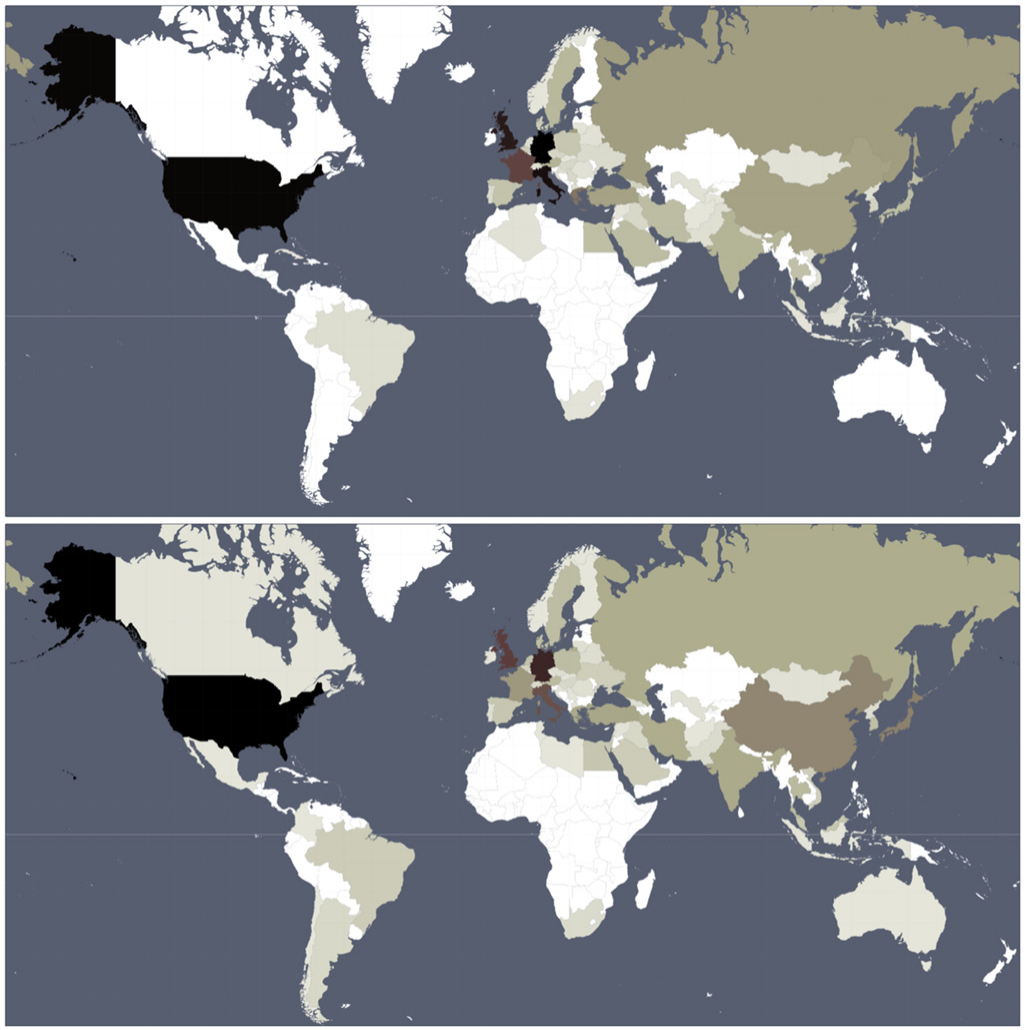
Sum of appearances of historical figures from a given country in the 24 lists of top 100 persons for PageRank (top panel) and 2DRank (bottom panel). Color changes from zero (white) to maximum (black). Maximal values are 233 appearances for Germany (top) and 236 for USA (bottom). Values are proportional to the averages per country shown in Fig. 1.

In Fig. 2 we show the world map of countries, where color indicates the number of persons from a given country among the 24 × 100 top persons for PageRank and 2DRank. Additional figures showing these distributions for different centuries are available at [39].

**Fig 3:**
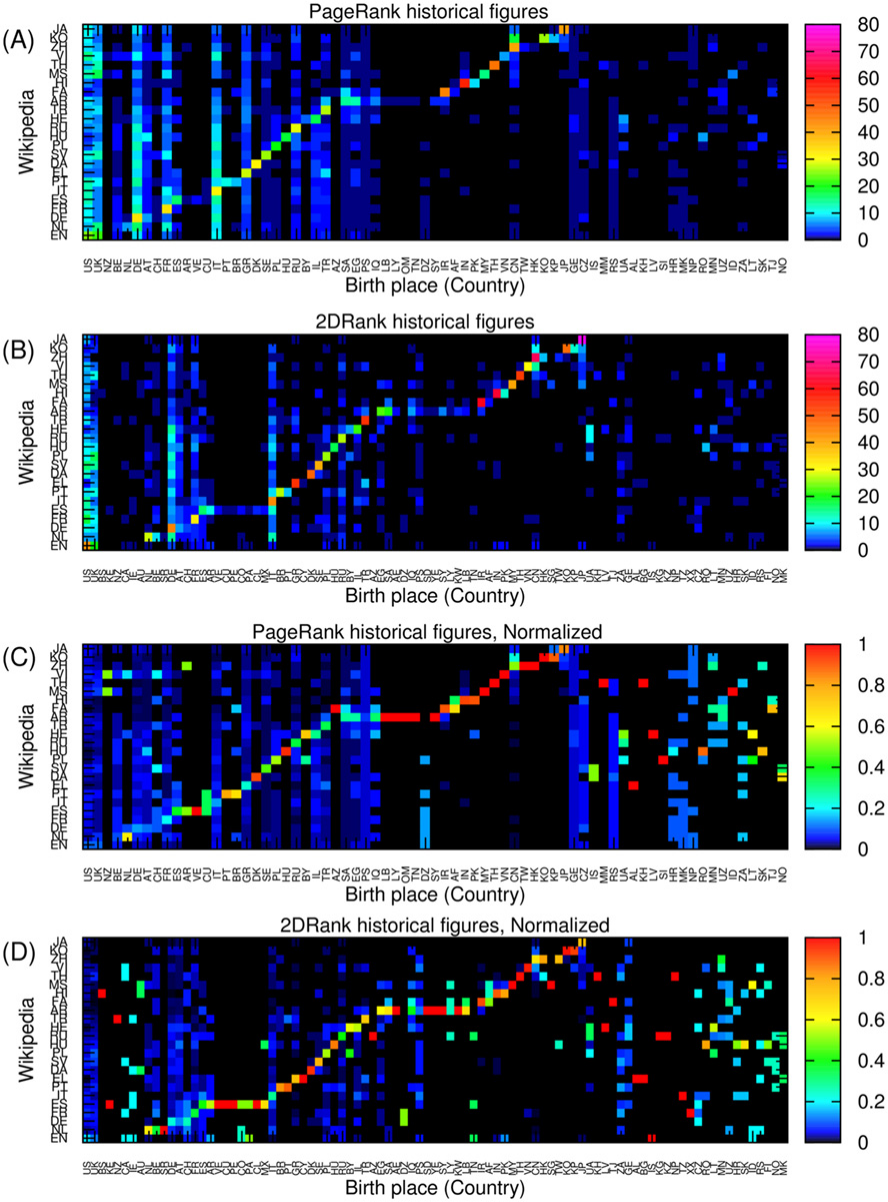
Birth place distributions over countries of top historical figures from each Wikipedia edition; two letter country codes are represented in Table 3. Panels: (A) distributions of PageRank historical figures over 71 countries for each Wikipedia edition; (B) distributions of 2DRank historical figures over 91 countries for each Wikipedia edition; (C) column normalized birth place distributions of PageRank historical figures of panel (A); (D) column normalized birth place distributions of 2DRank historical figures of panel (B).

We also observed local skewness in the spatial distribution of the top historical figures for the PageRank (2DRank) ranking algorithm as shown in Fig. 3A (in Fig. 3B). For example, 47 percent of the top PageRank historical figures in the English Wikipedia were born in USA (25 percent) and UK (22 percent) and 56 percent of the top historical figures in the Hindi Wikipedia were born in India. A similar strong locality pattern of the top historical figures was observed in our previous research [21]. However it should be noted that in the previous study we considered the native language of the top historical figure as a criterion of locality, while in the current study we considered ‘birth place’ as criterion of locality.

Regional skewness, the preferences of Wikipedia editions for historical figures who were born in geographically or culturally related countries, is also observed. For example, 18 (5) of the top 100 PageRank historical figures in the Korean (Japanese) Wikipedia were born in China. Also 9 of the top 100 PageRank historical figures in the Persian Wikipedia were born in Saudi Arabia. The distribution of top persons from each Wikipedia edition over world countries is shown in Fig. 3A and Fig. 3B. The countries on a horizontal axis are grouped by clusters of corresponding language so that the links inside a given culture (or language) become well visible.

To observe patterns in a better way at low numbers of historical figures, we normalized each column of Fig. 3A and Fig. 3B corresponding to a given country. In this way we obtain a rescaled distribution with better visibility for each birth country level as shown in Fig. 3C and Fig. 3D, respectively. We can observe a clear birth pattern of top PageRank historical figures born in Lebanon, Libya, Oman, and Tunisia in the case of the Arabic Wikipedia, and historical figures born in N. Korea appearing not only in the Korean but also in the Japanese Wikipedia.

In the case of the top 2DRank historical figures shown in Fig. 3B and Fig. 3D, we observe overall patterns of locality and regions being similar to the case of PageRank, but the locality is stronger.

In short, we observed that most of the top historical figures in Wikipedia were born in Western countries, but also that each edition shows its own preference to the historical figures born in countries which are closely related to the corresponding language edition.

### Temporal distribution

**Fig 4:**
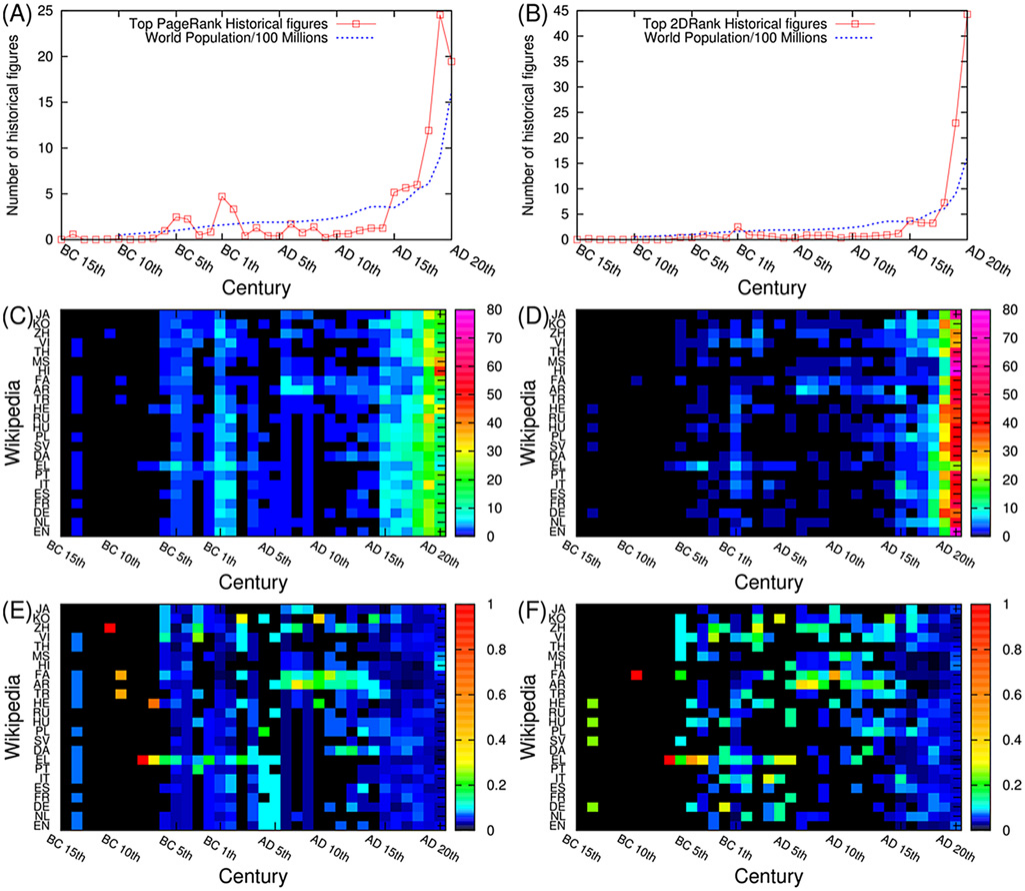
Birth date distributions of top historical figures. (A) Birth date distribution of PageRank historical figures averaged over 24 Wikipedia editions (B) Birth date distribution of 2DRank historical figures averaged over 24 Wikipedia editions (C) Birth date distributions of PageRank historical figures for each Wikipedia edition. (D) Birth date distributions of 2DRank historical figures for each Wikipedia edition. (E) Column normalized birth date distributions of PageRank historical figures for each Wikipedia edition. (F) Column normalized birth date distributions of 2DRank historical figures for each Wikipedia edition.

The analysis of the temporal distribution of top historical figures is done based on their birth dates. As shown in Fig. 4A for PageRank, most of historical figures were born after the 17th century on average, which shows similar pattern with world population growth [40]. However, there are some distinctive peaks around BC 5th century and BC 1st century for the case of PageRank because of Greek scholars (*Socrates, Plato*, and *Herodotus*), Roman politicians (*Julius Caesar, Augustus*) and Christianity leaders (*Jesus, Paul the Apostle*, and *Mary (mother of Jesus)*). We also observe that the Arabic and the Persian Wikipedia have more historical figures than Western language Wikipedia editions from AD 6th century to AD 12th century. For the case of 2DRank in Fig. 4B, there is only one small peak around BC 1C, which is also smaller than the peak in the case of PageRank, and all the distribution is dominated by a strong growth on the 20th century.

The distributions of the top PageRank historical figures over the 24 Wikipedia editions for each century are shown in Fig. 4C. The same distribution, but normalized to unity over all editions for each century, is shown in Fig. 4E. The Persian (FA) and the Arabic (AR) Wikipedia have more historical figures than other language editions (in particular European language editions) from the 6th to the 12th century due to Islamic leaders and scholars. On the other hand, the Greek Wikipedia has more historical figures in BC 5th century because of Greek philosophers. Also most of western-southern European language editions, including English, Dutch, German, French, Spanish, Italian, Portuguese, and Greek, have more top historical figures because they have *Augustine the Hippo* and *Justinian I* in common. Similar distributions obtained from 2DRank are shown in Fig. 4D and Fig. 4F respectively.

The data of Figs. 4E, F clearly show well pronounced patterns, corresponding to strong interactions between cultures: from BC 5th century to AD 15th century for JA, KO, ZH, VI; from AD 6th century to AD 12th century for FA, AR; and a common birth pattern in EN, EL, PT, IT, ES, DE, NL (Western European languages) from BC 5th century to AD 6th century. In supporting Figure S1 we show distributions of historical figures over languages according to their birth place. In this case the above patterns become even more pronounced.

**Fig 5:**
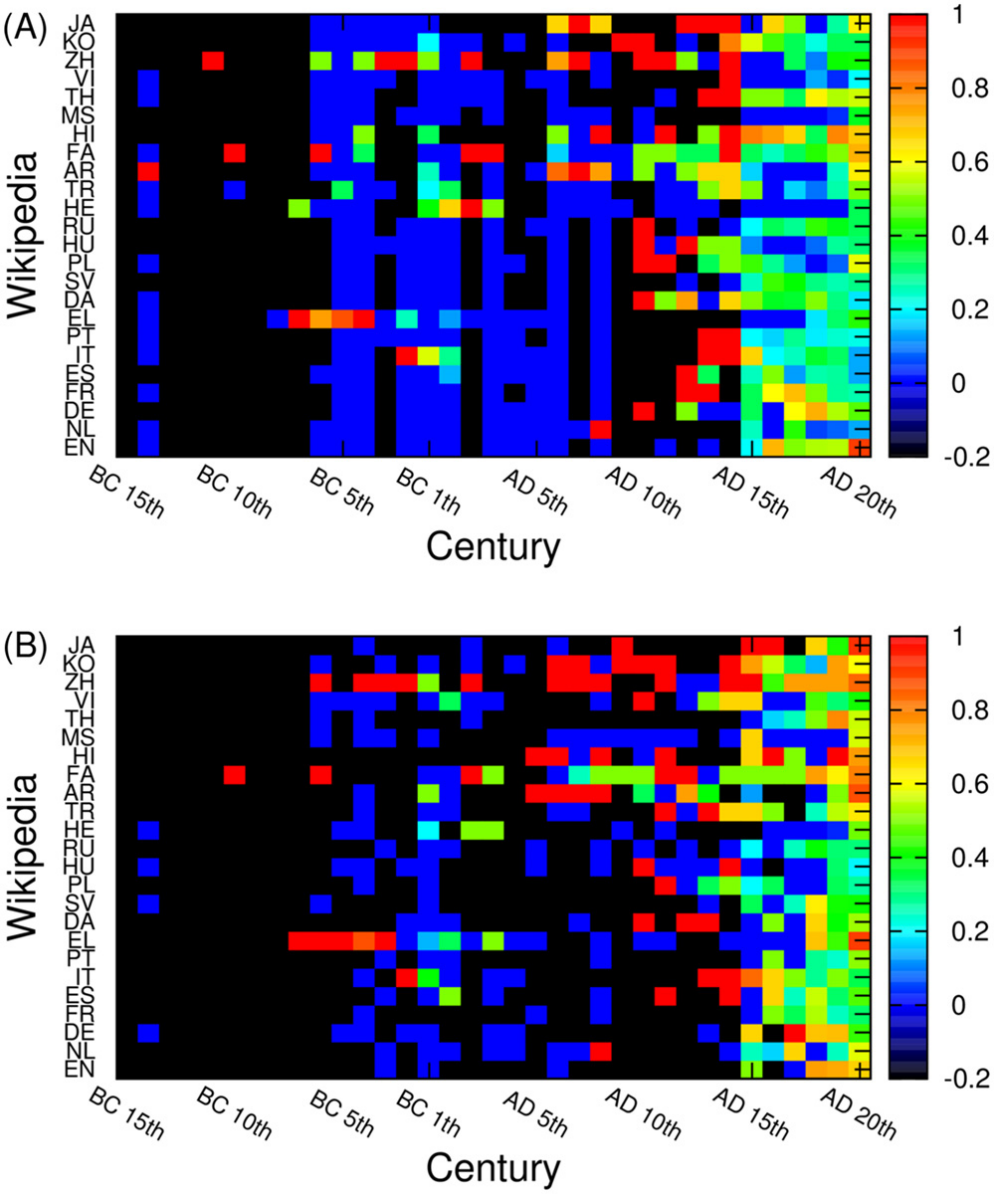
The locality property of cultures represented by the ratio *r*_*L*, *C*_ = *M*_*L*, *C*_/*N*_*L*, *C*_ for each edition *L* and each century *C*. Here *M*_*L*, *C*_ is the number of historical figures born in countries attributed to a given language edition *L* at century *C* and *N*_*L*, *C*_ is the total number of historical figures in a given edition at a given century, regardless of language of their birth countries. Black color (-0.2 in the color bars) shows that there is no historical figure at all for a given edition and century; blue (0 in the color bars) shows there there are some historical figures but no same language historical figures. Here (A) panel shows PageRank historical figures, and (B) panel shows 2DRank historical figures.

At a first glance from Figs. 4E, F we observe for persons born in AD 20th century a significantly more homogeneous distribution over cultures compared to early centuries. However, as noted in [21], each Wikipedia edition favors historical figures speaking the corresponding language. We investigate how this preference to same-language historical figures changes in time. For this analysis, we define two variables *M*_*L*, *C*_ and *N*_*L*, *C*_ for a given language edition *L* and a given century *C*. Here *M*_*L*, *C*_ is the number of historical figures born in all countries being attributed to a given language *L*, and *N*_*L*, *C*_ is the total number of historical figures for a given century *C* and a given language edition *L*. For example, among the 21 top PageRank historical figures from the English Wikipedia, who were born in AD 20th century, two historical figures (Pope John Paul II and Pope Benedict XVI) were not born in English speaking countries. Thus in this case *N*_*EN*, 20_ = 21 and *M*_*EN*, 20_ = 19. Fig. 5 represents the ratio *r*_*L*, *C*_ = *M*_*L*, *C*_/*N*_*L*, *C*_ for each edition and each century. In ancient times (i.e. before AD 5th century), most historical figures for each Wikipedia edition are not born in the same language region except for the Greek, Italian, Hebrew, and Chinese Wikipedia. However, after AD 5th century, the ratio of same language historical figures is rising. Thus, in AD 20th century, most Wikipedia editions have significant numbers of historical figures born in countries speaking the corresponding language. For PageRank persons and AD 20th century, we find that the English edition has the largest fraction of its own language, followed by Arabic and Persian editions while other editions have significantly large connections with other cultures. For the English edition this is related to a significant number of USA presidents appearing in the top 100 list (see [18, 19]). For 2DRank persons the largest fractions were found for Greek, Arabic, Chinese and Japanese cultures. These data show that even in age of globalization there is a significant dominance of local historical figures for certain cultures.

### Gender distribution

**Fig 6:**
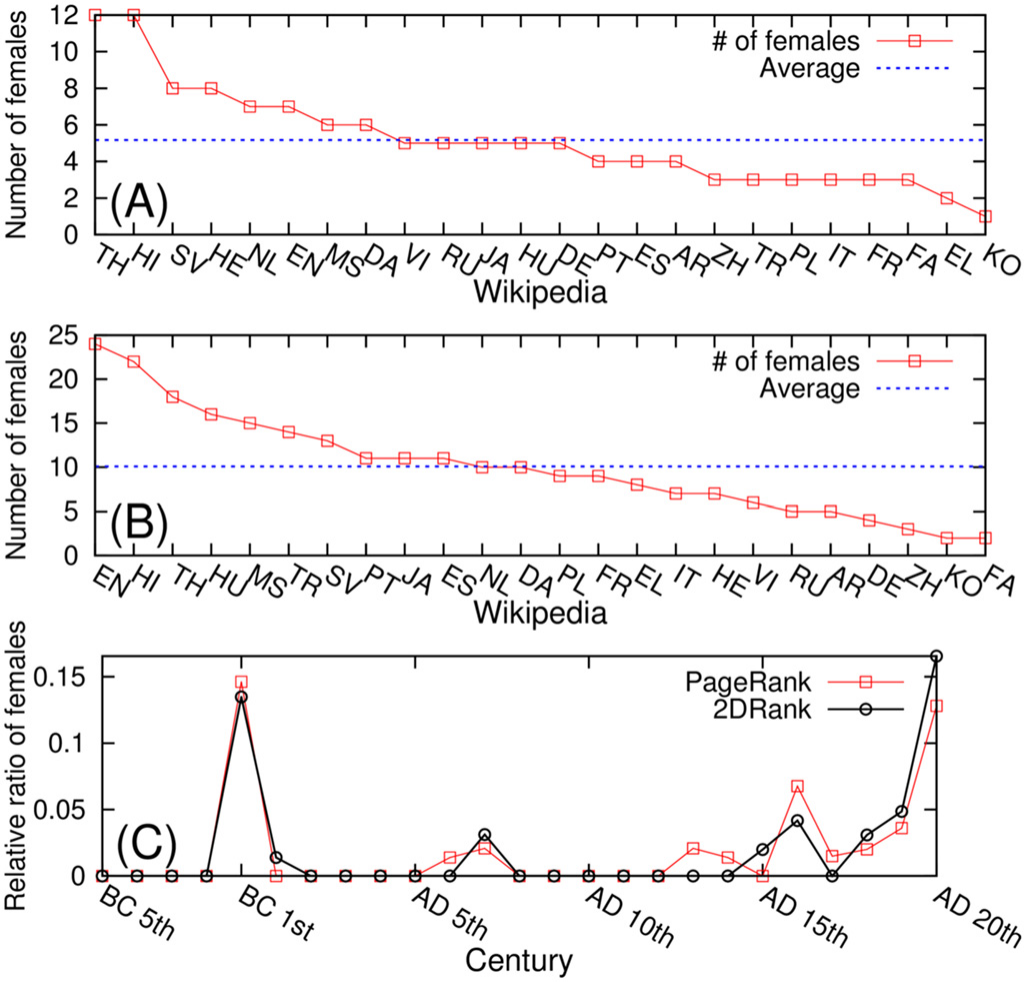
Number of females of top historical figures from each Wikipedia edition (A) Top PageRank historical figures (B) Top 2DRank historical figures. (C) The average female ratio of historical figures in given centuries across 24 Wikipedia editions.

From the gender distributions of historical figures, we observe a strong male-skewed pattern across many Wikipedia editions regardless of the ranking algorithm. On average, 5.2(10.1) female historical figures are observed among the 100 top PageRank (2DRank) persons for each Wikipedia edition. Fig. 6 shows the number of top female historical figures for each Wikipedia edition. Thai, Hindi, Swedish, and Hebrew have more female historical figures than the average over our 24 editions in the case of PageRank. On the other hand, the Greek and the Korean versions have a lower number of females than the average. In the case of 2DRank, English, Hindi, Thai, and Hungarian Wikipedia have more females than the average while German, Chinese, Korean, and Persian Wikipedia have less females than the average. In short, the top historical figures in Wikipedia are quite male-skewed. This is not surprising since females had little chance to be historical figures for most of human history. We compare the gender skewness to other cases such as the number of female editors in Wikipedia (9 percent) in 2011 [41] and the share of women in parliaments, which was 18.7 percent in 2012 by UN Statistics and indicators on women and men [42], the male skewness for the PageRank list is stronger in the contents of Wikipedia [43]. However, the ratio of females among the top historical figures is growing by time as shown in Fig. 6 C. It is notable that the peak in Fig. 6C at BC 1st is due to “Mary (mother of Jesus)”. In the 20th century 2DRank gives a larger percentage of women compared to PageRank. This is due to the fact that 2DRank has a larger fraction of singers and artists comparing to PageRank (see [18, 19]) and that the fraction of women in these fields of activity is larger.

### Global historical figures

Above we analyzed how top historical figures in Wikipedia are distributed in terms of space, time, and gender. Now we identify how these top historical figures are distributed in each Wikipedia edition and which are global historical figures. According to previous research [21], there are some global historical figures who are recognized as important historical figures across Wikipedia editions. We identify global historical figures based on the ranking score for a given person determined by her number of appearances and ranking index over our 24 Wikipedia editions.

Following [21], the ranking score Θ_*P*, *A*_ of a historical figure *P* is given by

$$\begin{array}{c} \Theta_{P,A}=\sum_{E}\left(101-R_{P,E,A}\right) \end{array}$$ (3)

Here *R*_*P*, *E*, *A*_ is the ranking of a historical figure *P* in Wikipedia edition *E* by ranking algorithm *A*. According to this definition, a historical figure who appears more often in the lists of top historical figures for the given 24 Wikipedia editions or has higher ranking in the lists gets a higher ranking score. Table 4 represents the top 10 global historical figures for PageRank and 2DRank. *Carl Linnaeus* is the 1st global historical figure by PageRank followed by *Jesus, Aristotle. Adolf Hitler* is the 1st global historical figure by 2DRank followed by *Michael Jackson, Madonna (entertainer)*. On the other hand, the lists of the top 10 local historical figures ordered by our ranking score for each language are represented in supporting Tables S1–S25 and [39].

**Table 4:** List of global historical figures by PageRank and 2DRank for all 24 Wikipedia editions. All names are represented by the corresponding article titles in the English Wikipedia. Here, Θ_*A*_ is the ranking score of algorithm *A* (3); *N*_*A*_ is the number of appearances of a given person in the top 100 rank for all editions.

**Rank**	**PageRank global figures**	**Θ_*PR*_**	***N*_*A*_**	**2DRank global figures**	**Θ_2*D*_**	***N*_*A*_**
1st	Carl Linnaeus	2284	24	Adolf Hitler	1557	20
2nd	Jesus	2282	24	Michael Jackson	1315	17
3rd	Aristotle	2237	24	Madonna (entertainer)	991	14
4th	Napoleon	2208	24	Jesus	943	14
5th	Adolf Hitler	2112	24	Ludwig van Beethoven	872	14
6th	Julius Caesar	1952	23	Wolfgang Amadeus Mozart	853	11
7th	Plato	1949	24	Pope Benedict XVI	840	12
8th	William Shakespeare	1861	24	Alexander the Great	789	11
9th	Albert Eistein	1847	24	Charles Darwin	773	12
10th	Elizabeth II	1789	24	Barack Obama	754	16

The reason for a somewhat unexpected PageRank leader *Carl Linnaeus* is related to the fact that he laid the foundations for the modern biological naming scheme so that plenty of articles about animals, insects and plants point to the Wikipedia article about him, which strongly increases the PageRank probability. This happens for all 24 languages where *Carl Linnaeus* always appears on high positions since articles about animals and plants are an important fraction of Wikipedia. Even if in a given language the top persons are often politicians (e.g. *Napoleon, Barak Obama* at *K* = 1, 2 in EN), these politicians have mainly local importance and are not highly ranked in other languages (e.g. in ZH *Carl Linnaeus* is at *K* = 1, *Napoleon* at *K* = 3 and *Barak Obama* is at *K* = 24). As a result when the global contribution is counted over all 24 languages *Carl Linnaeus* appears on the top PageRank position.

**Fig 7:**
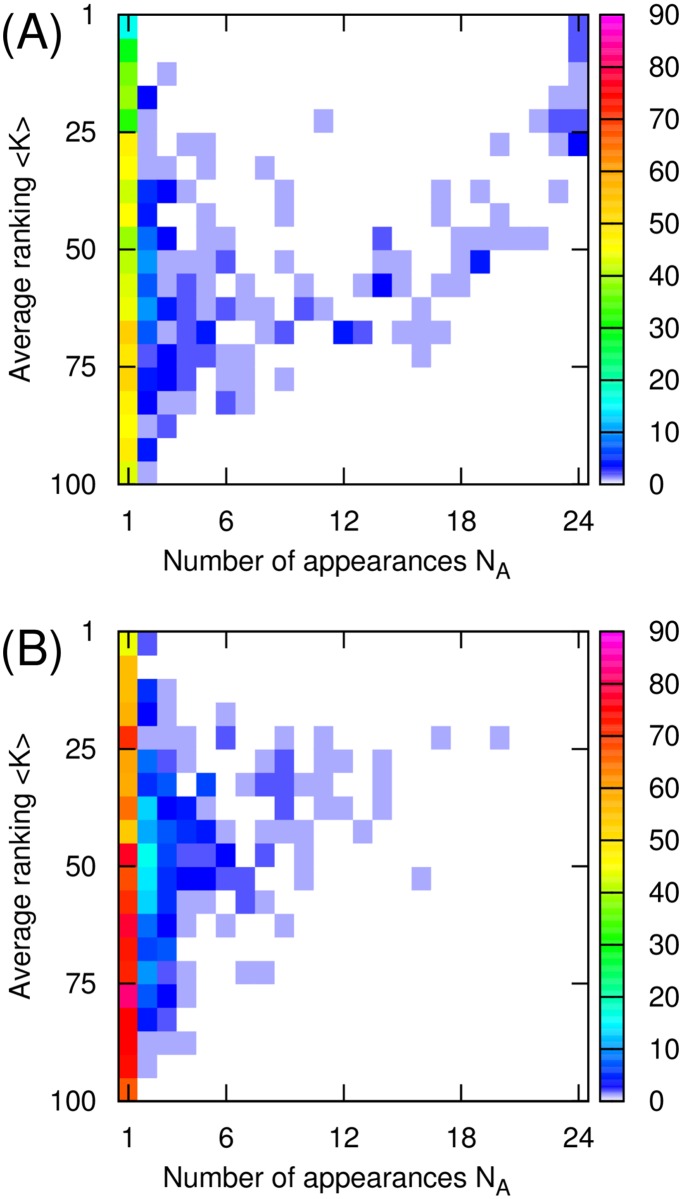
The distribution of 1045 top PageRank persons (A) and 1616 top 2DRank persons (B) as a function of number of appearances *N*_*A*_ of a given person and the rank ⟨*K*⟩ of this person averaged over Wikipedia editions where this person appeared.

Our analysis suggests that there might be three groups of historical figures. Fig. 7 shows these three groups of top PageRank historical figures in Wikipedia: (i) global historical figures who appear in most of Wikipedia editions (*N*_*A*_ ≥ 18) and are highly ranked (⟨*K*⟩ ≤ 50) for each Wikipedia such as Carl Linnaeus, Plato, Jesus, and Napoleon (Right-Top of the Fig. 7A); (ii) local-highly ranked historical figures who appear in a few Wikipedia editions (*N*_*A*_ < 18) but are highly ranked (⟨*K*⟩ ≤ 50) in the Wikipedia editions in which they appear, such as Tycho Brahe, Sejong the Great, and Sun Yat-sen (Left-Top of the Fig. 7A); (iii) locally-low ranked historical figures who appear in a few Wikipedia editions (*N*_*A*_ < 18) and who are not highly ranked (⟨*K*⟩ > 50). Here *N*_*A*_ is the number of appearances in different Wikipedia editions for a given person and ⟨*K*⟩ is the average ranking of the given persons across Wikipedia editions for each ranking algorithm. In the case of 2DRank historical figures, due to the absence of global historical figures, most of them belong to two types of local historical figures (i.e. local-highly ranked or local-lowly ranked).

Following ranking of persons via Θ_*P*, *A*_ we determine also the top global female historical figures, presented in Table 5 for PageRank and 2DRank persons. The full lists of global female figures are available at [39] (63 and 165 names for PageRank and 2DRank).

**Table 5:** List of the top 10 global female historical figures by PageRank and 2DRank for all the 24 Wikipedia editions. All names are represented by article titles in the English Wikipedia. Here, Θ_*A*_ is the ranking score of the algorithm *A* (Eq.3); *N*_*A*_ is the number of appearances of a given person in the top 100 rank for all editions. Here *CC* is the birth country code and *LC* is the language code of the given historical figure.

**Rank**	**Θ_*PR*_**	***N*_*A*_**	**PageRank female figures**	***CC***	**Century**	***LC***
1	1789	24	Elizabeth II	UK	20	EN
2	1094	17	Mary (mother of Jesus)	IL	-1	HE
3	404	12	Queen Victoria	UK	19	EN
4	234	6	Elizabeth I of England	UK	16	EN
5	128	2	Maria Theresa	AT	18	DE
6	100	1	Benazir Bhutto	PK	20	HI
7	94	1	Catherine the Great	PL	18	PL
8	91	1	Anne Frank	DE	20	DE
9	87	1	Indira Gandhi	IN	20	HI
10	86	1	Margrethe II of Denmark	DK	20	DA

**Rank**	**Θ_2*D*_**	***N*_*A*_**	**2DRank female figures**	***CC***	**Century**	***LC***
1	991	14	Madonna (entertainer)	US	20	EN
2	664	9	Elizabeth II	UK	20	EN
3	580	8	Mary (mother of Jesus)	IL	-1	HE
4	550	9	Queen Victoria	UK	19	EN
5	225	5	Agatha Christie	UK	19	EN
6	211	4	Mariah Carey	US	20	EN
7	206	7	Britney Spears	US	20	EN
8	200	3	Margaret Thatcher	UK	20	EN
9	191	2	Martina Navratilova	CZ	20	WR
10	175	2	Elizabeth I of England	UK	16	EN

The comparison of our 100 global historical figures with the top 100 from Hart’s list [27] gives an overlap of 43 persons for PageRank and 26 persons for 2DRank. We note that for the top 100 from the English Wikipedia we obtain a lower overlap of 37 (PageRank) and 4 (2DRank) persons. Among all editions the highest overlaps with the Hart list are 42 (VI), 37 (EN, ES, PT, TR) and 33 (IT), 32 (DE), 31 (FR) for PageRank; while for 2DRank we find 18 (EL) and 17 (VI). We give the overlap numbers for all editions at [39]. This shows that the consideration of 24 editions provides us the global list of the top 100 persons with a more balanced selection of top historical figures. Our overlap of the top 100 global historical figures by PageRank with the top 100 people from Pantheon MIT ranking list [23] is 44 percent, while the overlap of this Pantheon list with Hart’s list is 43 percent. We note that the Pantheon method is significantly based on a number of page views while our approach is based on the network structure of the whole Wikipedia network. The top 100 persons from [22] are not publicly available but nevertheless we present the overlaps between the top 100 persons from the lists of Hart, Pantheon, Stony-Brook and our global PageRank and 2DRank lists in Figures S2, S3 (we received the Stony-Brook list as a private message from the authors of [22]). We have an average overlap between the 4 methods on a level of 40 percent (2DRank is on average lower by a few percent), we find a larger overlap between our PageRank list and the Stony-Brook list since the Stony-Brook method, applied only for the English Wikipedia, is significantly based on PageRank.

We also compared the distributions of our global top 100 persons of PageRank and 2DRank with the distribution of Hart’s top 100 over centuries and over 24 languages with the additional WR category (see Figure S4). We find that these 3 distributions have very similar shapes. Thus the largest number of persons appears in centuries AD 18th, 19th, 20th for the 3 distributions. Among languages, the main peaks for the 3 distributions appear for EN, DE, IT, EL, AR, ZH. The deviations from Hart’s distribution are larger for the 2DRank list. Thus the comparison of distributions over centuries and languages shows that the PageRank list has not only a strong overlap with the Hart list in the number of persons but that they also have very similar statistical distributions of the top 100 persons over centuries and languages.

The overlap of the top 100 global persons found here with the previous study [21] gives 54 and 47 percent for PageRank and 2DRank lists, respectively. However, we note that the global list in [21] was obtained from the top 30 persons in each edition while here we use the top 100 persons.

It is interesting to note that for the top 100 PageRank universities from the English Wikipedia edition the overlap with Shanghai top 100 list of universities is on a even higher level of 75 percent [18].

Finally, we note that the ranking of historical figures using the whole PageRank (or 2DRank) list of all Wikipedia articles of a given edition provides a more stable approach compared to the network of biographical articles used in [20]. Indeed, the number of nodes and links in such a biographical network is significantly smaller compared to the whole network of Wikipedia articles and thus the fluctuations become rather large. For example, from the biographical network of the Russian edition one finds as the top person *Napoleon III* (and even not *Napoleon I*) [20], who has a rather low importance for Russia. In contrast to that the present study gives us the top PageRank historical figure of the Russian edition to be *Peter the Great*, that has much more historical grounds. In a similar way for FR the results of [20] give at the first position *Adolf Hitler*, that is rather strange for the French culture, while we find a natural result *Napoleon*.

### Network of cultures

We consider the selected top persons from each Wikipedia edition as important historical figures recognized by people who speak the language of that Wikipedia edition. Therefore, if a top person from a language edition *A* appears in another edition *B*, then we can consider this as a ‘cultural’ influence from culture *A* to *B*. Here we consider each language as a proxy for a cultural group and assign each historical figure to one of these cultural groups based on the most spoken language of her/his birth place at the country level. For example, *Adolf Hitler* was born in modern Austria and since German language is the most spoken language in Austria, he is considered as a German historical figure in our analysis. This method may lead to some misguiding results due to discrepancy between territories of country and cultures, e.g. *Jesus* was born in the modern State of Palestine (Bethlehem), which is an Arabic speaking country. Thus *Jesus* is from the Arabic culture in our analysis while usually one would say that he belongs to the Hebrew culture. Other similar examples we find are: *Charlemagne* (Belgium—Dutch), *Immanuel Kant* (Russia—Russian, while usually he is attributed to DE), *Moses* (Egypt—Arabic), *Catherine the Great* (Poland—Polish, while usually she would be attributed to DE or RU).

In total there are such 36 cases from the global PageRank list of 1045 names (these 36 names are given in SI). However, in our knowledge, the birth place is the best way to assign a given historical figure to a certain cultural background computationally and systematically and with the data we have available. In total we have only about 3.4 percent of cases which can be discussed and where a native speaking language can be a better indicator of belonging to a given culture. For the global 2DRank list of 1616 names we identified 53 similar cases where an attribution to a culture via a native language or a birth place could be discussed (about 3.3 percent). These 53 names are given in SI. About half of such cases are linked with birth places in ancient Russian Empire where people from Belarus, Litvania and Ukraine moved to RU, IL, PL, WR. However, the percentage of such cases is small and the corresponding errors also remain small.

Based on the above assumption and following the approach developed in [21], we construct two weighted networks of cultures (or language groups) based on the top PageRank historical figures and top 2DRank historical figures respectively. Each culture (i.e. language) is represented as a node of the network, and the weight of a directed link from culture *A* to culture *B* is given by the number of historical figures belonging to culture *B* (e.g. French) appearing in the list of top 100 historical figures for a given culture *A* (e.g. English). The persons in a given edition, belonging to the language of the edition, are not taken into account since they do not create links between cultures. In Table 6 we give the number of such persons for each language. This table also gives the number of persons of a given language among the top 100 persons of the global PageRank and 2DRank listings.

**Table 6:** Numbers of certain historical figures for top 100 list of each language: *N*_1_ is the number of historical figures of a given language among the top 100 PageRank global historical figures; *N*_2_ is the number of historical figures of a given language among the top 100 PageRank historical figures for the given language edition; *N*_3_ is the number of historical figures of a given language among the top 100 2DRank global historical figures; *N*_4_ is the number of historical figures of a given language among the top 100 2DRank historical figures for the given language edition.

**Language**	***N*_1_**	***N*_2_**	***N*_3_**	***N*_4_**	**Language**	***N*_1_**	***N*_2_**	***N*_3_**	***N*_4_**
EN	22	47	27	64	RU	2	29	3	27
NL	2	10	4	38	HE	2	17	2	22
DE	20	41	16	55	TR	2	27	2	54
FR	8	33	3	32	AR	8	42	5	69
ES	2	20	5	39	FA	0	46	1	64
IT	11	31	9	43	HI	1	65	0	76
PT	0	19	0	35	MS	0	15	0	40
EL	5	28	2	55	TH	0	46	0	53
DA	0	31	1	48	VI	0	7	0	30
SV	1	26	1	39	ZH	5	43	6	79
PL	1	20	2	26	KO	0	34	0	59
HU	0	18	0	18	JA	0	41	4	80
WR	8	-	7	-					

**Fig 8:**
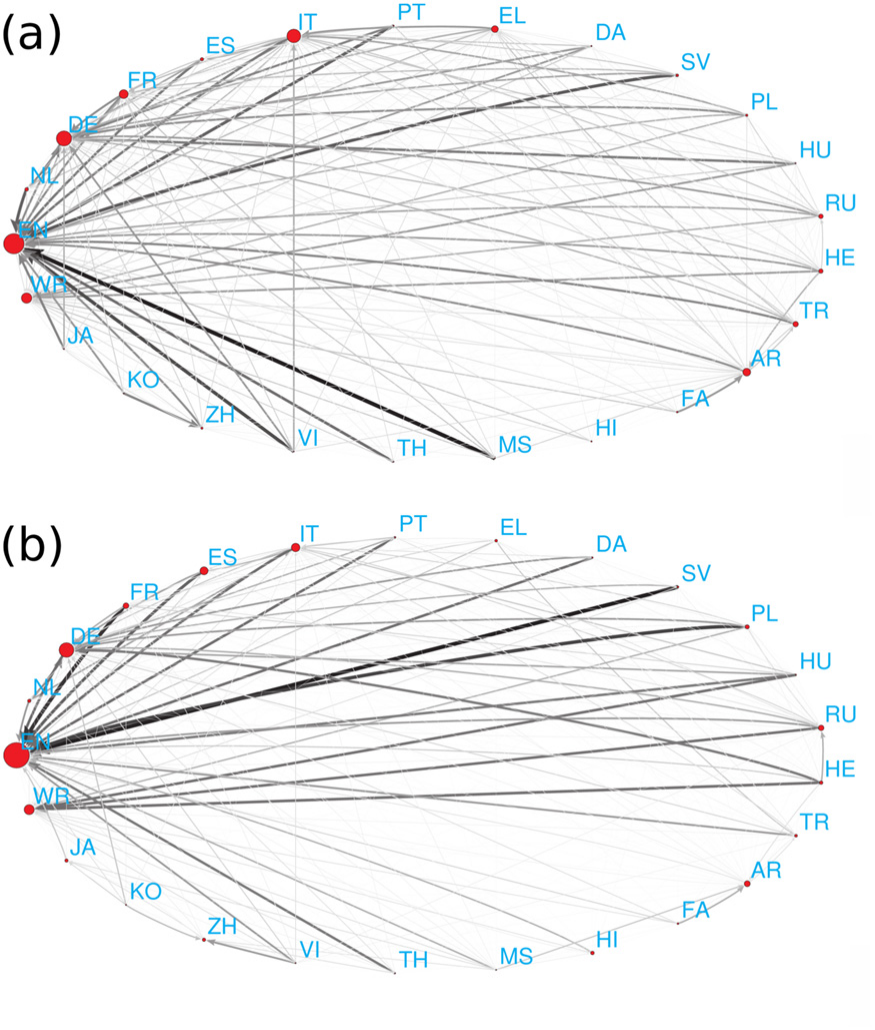
Network of cultures obtained from 24 Wikipedia languages and the remaining world (WR) consider (A) top PageRank historical figures and (B) 2DRank historical figures. The link width and darkness are proportional to a number of foreign historical figures quoted in top 100 of a given culture, the link direction goes from a given culture to cultures of quoted foreign historical figures, links inside cultures are not considered. The size of nodes is proportional to their PageRank.

For example, there are 5 French historical figures among the top 100 PageRank historical figures of the English Wikipedia, so we can assign weight 5 to the link from English to French. Fig. 8A and Fig. 8B represent the constructed networks of cultures defined by appearances of the top PageRank historical figures and top 2DRank historical figures, respectively. In total we have two networks with 25 nodes which include our 24 editions and an additional node WR for all the other world cultures.

**Fig 9:**
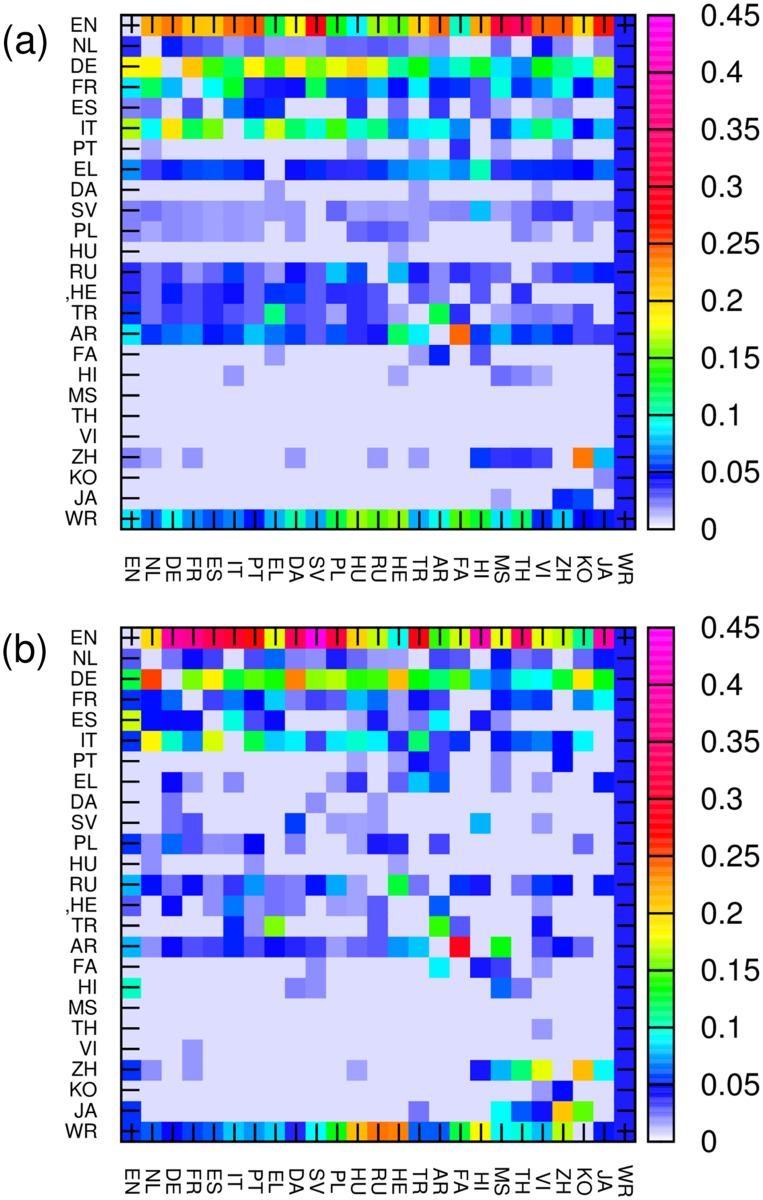
Google matrix of network of cultures shown in Fig. 8 respectively. The matrix elements *G*_*ij*_ are shown by color with damping factor *α* = 0.85.

The Google matrix *G*_*ij*_ for each network is constructed following the standard rules described in [21] and in the Methods Section. In a standard way we determine the PageRank index *K* and the CheiRank index K* that order all cultures according to decreasing PageRank and CheiRank probabilities (see Methods and Figure S5). The structure of matrix elements *G*_*KK*^′^_ is shown in Fig. 9.

**Fig 10:**
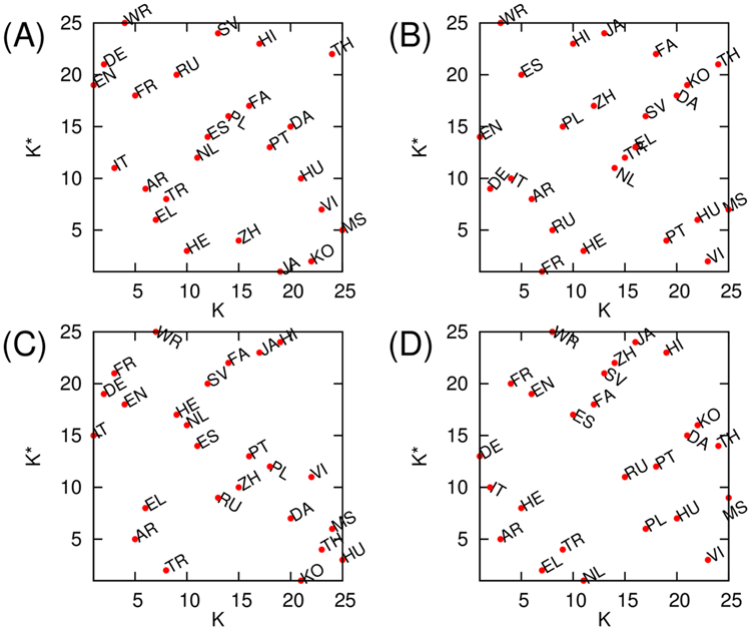
PageRank ranking versus CheiRank ranking plane of cultures with corresponding indexes *K* and K* obtained from the network of cultures based on (A) all PageRank historical figures, (B) all 2DRank historical figures, (C) PageRank historical figure born before AD 19th century, and (D) 2DRank historical figure born before AD 19th century, respectively.

To identify which cultures (or language groups) are more influential than others, we calculated PageRank and CheiRank of the constructed networks of cultures by considering link weights. Briefly speaking, a culture has high PageRank (CheiRank) if it has many ingoing (outgoing) links from (to) other cultures (see Methods). The distribution of cultures on a PageRank-CheiRank plane is shown in Fig. 10. In both cases of PageRank and 2DRank historical figures, historical figures of English culture (i.e. born in English language spoken countries) are the most influential (highest PageRank) and German culture is the second one (Fig. 10A, B). Here we consider the historical figures for the whole range of centuries. Fig. 10 represents the detailed features of how each culture is located on the plane of PageRank ranking *K* and CheiRank ranking K* based on the top PageRank historical figures (Fig. 10A) and top 2DRank historical figures (Fig. 10B). Here *K* indicates the ranking of a given culture ordered by how many of its own top historical figures appear in other Wikipedia editions, and K* indicates the ranking of a given culture according to how many of the top historical figures in the considered culture are from other cultures. As described above, English is on (*K* = 1, K* = 19) and German is on (*K* = 2, K* = 21) in the case of PageRank historical figures (Fig. 10A). In the case of 2DRank historical figures, English is on (*K* = 1, K* = 14) and German is on (*K* = 2, K* = 9).

It is important to note that there is a significant difference compared to the previous study [21]: there, only 9 editions had been considered and the top positions were attributed to the world node WR which captured a significant fraction of the top persons. This indicated that 9 editions are not sufficient to cover the whole world. Now for 24 editions we see that the importance of the world node WR is much lower (it moves from *K* = 1 for 9 editions [21] to *K* = 4 and 3 in Fig. 10A and Fig. 10B). Thus our 24 editions cover the majority the world. Still it would be desirable to add a few additional editions (e.g. Ukraine, Baltic Republics, Serbia etc.) to fill certain gaps.

It is interesting to note that the ranking plane of cultures (*K*, K*) changes significantly in time. Indeed, if we take into account only persons born before the 19th century then the ranking is modified with EN going to 4th (Fig. 10C for PageRank figures) and 6th position (Fig. 10C for 2DRank figures) while the top positions are taken by IT, DE, FR and DE, IT, AR, respectively.

At the same time, we may also argue that for cultures it is important not only to be cited but also to be communicative with other cultures. To characterize communicative properties of nodes on the network of cultures shown in Fig. 8 we use again the concepts of PageRank, CheiRank and 2DRank for these networks as described in Methods and [21]. Thus, for the network of cultures of Fig. 8, the 2DRank index of cultures highlights their influence in a more balanced way taking into account their importance (incoming links) and communicative (outgoing links) properties in a balanced manner.

Thus we find for all centuries at the top positions Greek, Turkish and Arabic (for PageRank persons) and French, Russian and Arabic (for 2DRank persons). For historical figures before the 19th century, we find respectively Arabic, Turkish and Greek (for PageRank) and Arabic, Greek and Hebrew (for 2DRank). The high position of Turkish is due to its close links both with Greek culture in ancient times and with Arabic culture in more recent times. We see also that with time the positions of Greek in 2DRank improves due to a global improved ranking of Western cultures closely connected with Greece.

## Discussion

By investigating birth place, birth date, and gender of important historical figures determined by the network structure of Wikipedia, we identified spatial, temporal, and gender skewness in Wikipedia. Our analysis shows that the most important historical figures across Wikipedia language editions were born in Western countries after the 17th century, and are male. Also, each Wikipedia edition highlights local figures so that most of its own historical figures are born in the countries which use the language of the edition. The emergence of such pronounced accent to local figures seems to be natural since there are more links and interactions within one culture. This is also visible from to the fact that in many editions the main country for the given language is at the first PageRank position among all articles (e.g. Russia in RU edition) [21]. Despite such a locality feature, there are also global historical figures who appear in most of the considered Wikipedia editions with very high rankings. Based on the cross-cultural historical figures, who appear in multiple editions, we can construct a network of cultures which describes interactions and entanglement between cultures.

It is very difficult to describe history in an objective way and due to that it was argued that history is “an unending dialogue between the past and present” [44]. In a similar way we can say that history is an unending dialogue between different cultural groups.

We use a computational and data mining approach, based on rank vectors of the Google matrix of Wikipedia, to perform a statistical analysis of interactions and entanglement of cultures. We find that this approach can be used for selecting the most influential historical figures through an analysis of collectively generated links between articles on Wikipedia. Our results are coherent with studies conducted by historians [27], with an overlap of 43% of important historical figures. Thus, such a mathematical analysis of local and global historical figures can be a useful step towards the understanding of local and global history and interactions of world cultures. Our approach has some limitations, mainly caused by the data source and by the difficulty of defining culture boundaries across centuries. The ongoing improvement of structured content in Wikipedia through the WikiData project, eventually in conjunction with additional manual annotation, should allow to deal with these limitations. Furthermore, it would be useful to perform comparisons with other approaches to measure the interactions of cultures, such as the analysis of language crossings of multilingual users [45].

Influence of digital media on information dissemination and social collective opinions among the public is growing fast. Our research across Wikipedia language editions suggests a rigorous mathematical way, based on Markov chains and Google matrix, for the identification of important historical figures and for the analysis of interactions of cultures at different historical periods and in different world regions. We think that a further extension of this approach to a larger number of Wikipedia editions will provide a more detailed and balanced analysis of interactions of world cultures.

## Supporting Information

S1 File Supporting Information file S1 presents Figures S1–S5 with additional information discussed above in the main part of the paper, lists of top 100 global PageRank and 2DRank names; Tables S1–S25 of top 10 names of given language and remained world from the global PageRank and 2DRank ranking lists of persons ordered by the score Θ_*P*, *A*_ of Eq.(3). For a reader convenience the lists of all 100 ranked names for all 24 Wikipedia editions and corresponding network link data for each edition are also given at [39] in addition to Supporting Information file. All used computational data are publicly available at http://dumps.wikimedia.org/. All the raw data necessary to replicate the findings and conclusion of this study are within the paper, supporting information files and this Wikimedia web site.
(PDF)
