# Supplementary material for: Interactions of Cultures and Top People of Wikipedia from Ranking of 24 Language Editions
Source: PLoS One. 2015 Mar 4;10(3):e0114825. doi: 10.1371/journal.pone.0114825 (PMC4349893; doi:10.1371/journal.pone.0114825)
Supplement: S1 File — For a reader convenience the lists of all 100 ranked names for all 24 Wikipedia editions and corresponding network link data for each edition are also given at [39] in addition to Supporting Information file. All used computational data are publicly available at http://dumps.wikimedia.org/. All the raw data necessary to replicate the findings and conclusion of this study are within the paper, supporting information files and this Wikimedia web site. (PDF) [file pone.0114825.s001.pdf]

SUPPORTING INFORMATION FOR: Interactions of cultures and top people of Wikipedia from ranking of 24 language editions

Young-Ho Eom[1], Pablo Aragón[2], David Laniado[2], Andreas Kaltenbrunner[2], Sebastiano Vigna[3], Dima L. Shepelyansky[1,*]

1 *Laboratoire de Physique Théorique du CNRS, IRSAMC, Université de Toulouse, UPS, F-31062 Toulouse, France*

2 *Barcelona Media Foundation, Barcelona, Spain*

3 *Dipartimento di Informatica, Università degli Studi di Milano, Milano, Italy*

∗ Corresponding Author E-mail: dima@irsamc.ups-tlse.fr

1 Additional data

Here we present additional figures and tables for the main part of the paper.

Figure S1 is analogous to Figures 4(C,D,E,F), however, now on the vertical axis we plot not the edition to which a given historical figure is attributed from top 100 figures of a given edition but the language, to which this historical figure from the global PageRank (1045 persons) or 2DRank (1616 persons) lists is attributed according to our procedure according to her/his country of birth and then to the major language of this country, if a person does not belong to any of 24 languages then he/she is attributed to the remaining world (WR). The data show that the separation between language (or culture) groups becomes now more distinct. Indeed, attribution to a language related to a birth place is more definite compared to the option where a person appears in one of 24 editions since some global historical figures appear in a few editions while each person is attributed only one language according to our procedure.

Figure S2 shows overlap between the global list of top 100 global PageRank persons and list of Hart [23], PageRank list of English Wikipedia from [15], list of Stony-Brook [19], list of Pantheon MIT project [20].

Figure S3 shows the overlap matrix (in percent) between 5 methods of ranking of top 100 historical figures including Hart, Pantheon, Stony-Brook results and our global PageRank and 2DRank lists. We see that our PageRank has most high correlation with Stony-Brook since the method of Stony-Brook uses significantly the PageRank method.

Figure S4 shows the number of persons from top 100 lists of Hart and our global PageRank and 2DRank lists. The panel (A) shows the number of persons at a given century corresponding to the time dependence and the panel (B) shows distribution of such persons over the language they are attributed according to our method based on the birth place and dominant language of a country of birth. We see that the pattern of Hart ranking is well reproduced from our global ranking, especially for the case of PageRank list.

Figure S5 shows PageRank and CheiRank probabilities for the networks of cultures shown in Figure 8.

The names of persons from top 100 missed by automatic recovery of persons are: Homer, Charles Darwin (RU PageRank); Philipp Kirkorov (RU 2DRank); Alexander the Great, Emperor Gaozu of Han, Homer (KO PageRank); Jinpyeong of Silla, Hyeonjong of Goryeo (KO 2DRank).

Unfortunately, the name of Homer has been missed in the 1.1 million list of English names, other names are missed due to incompleteness and modifications of inter-language translations.

Below we give the list of global top 100 PageRank names from 24 Wikipedia editions. The names are ordered by the ranking score $\Theta_{P,A}$ of Eq.(1). In brackets we give country of birth, century of birth, gender, and language of birth. In the same manner we also give th list of top 100 2DRank names from 24 Wikipedia editions.

We also give 24 names from global 1045 PageRank names and 40 names from 1616 global 2DRank names where a birth place language attribution differs from native language.

We also give the tables of top 10 persons in each language and also world names (tables S1 - S25) extracted from the global PageRank and 2DRank ranking lists of persons ordered by the score $\Theta_{P,A}$ of Eq.(1).

Top 100 of global PageRank names: 1. Carl Linnaeus (SE, 18, M, SV) 2. Jesus (PS, -1, M, AR) 3. Aristotle (GR, -4, M, EL) 4. Napoleon (FR, 18, M, FR) 5. Adolf Hitler (AT, 19, M, DE) 6. Julius Caesar (IT, -1, M, IT) 7. Plato (GR, -5, M, EL) 8. William Shakespeare (UK, 16, M, EN) 9. Albert Einstein (DE, 19, M, DE) 10. Elizabeth II (UK, 20, F, EN) 11. Alexander the Great (GR, -4, M, EL) 12. Isaac Newton (UK, 17, M, EN) 13. Muhammad (SA, 6, M, AR) 14. Karl Marx (DE, 19, M, DE) 15. Joseph Stalin (GE, 19, M, WR) 16. Augustus (IT, -1, M, IT) 17. Christopher Columbus (IT, 15, M, IT) 18. Charlemagne (BE, 8, M, NL) 19. Louis XIV of France (FR, 17, M, FR) 20. George W. Bush (US, 20, M, EN) 21. Immanuel Kant (RU, 18, M, RU) 22. Barack Obama (US, 20, M, EN) 23. Mary (mother of Jesus) (IL, -1, F, HE) 24. Vladimir Lenin (RU, 19, M, RU) 25. Wolfgang Amadeus Mozart (AT, 18, M, DE) 26. Paul the Apostle (TR, 1, M, TR) 27. Charles Darwin (UK, 19, M, EN) 28. Martin Luther (DE, 15, M, DE) 29. Herodotus (TR, -5, M, TR) 30. Franklin D. Roosevelt (US, 19, M, EN) 31. Galileo Galilei (IT, 16, M, IT) 32. Pope John Paul II (PL, 20, M, PL) 33. Constantine the Great (RS, 3, M, WR) 34. Benito Mussolini (IT, 19, M, IT) 35. Cicero (IT, -2, M, IT) 36. Ren Descartes (FR, 16, M, FR) 37. Saint Peter (IL, 1, M, HE) 38. Ludwig van Beethoven (DE, 18, M, DE) 39. George Washington (US, 18, M, EN) 40. Moses (EG, -14, M, AR) 41. Johann Sebastian Bach (DE, 17, M, DE) 42. Bill Clinton (US, 20, M, EN) 43. Leonardo da Vinci (IT, 15, M, IT) 44. Johann Wolfgang von Goethe (DE, 18, M, DE) 45. Gautama Buddha (NP, -6, M, WR) 46. Winston Churchill (UK, 19, M, EN) 47. John F. Kennedy (US, 20, M, EN) 48. Charles V, Holy Roman Emperor (BE, 15, M, NL) 49. Pope Benedict XVI (DE, 20, M, DE) 50. Richard Nixon (US, 20, M, EN) 51. Sigmund Freud (CZ, 19, M, WR) 52. Ronald Reagan (US, 20, M, EN) 53. Abraham Lincoln (US, 19, M, EN) 54. Saddam Hussein (IQ, 20, M, AR) 55. Ptolemy (EG, 1, M, AR) 56. Richard Wagner (DE, 19, M, DE) 57. Diocletian (HR, 3, M, WR) 58. Queen Victoria (UK, 19, F, EN) 59. Napoleon III (FR, 19, M, FR) 60. Charles de Gaulle (FR, 19, M, FR) 61. Mao Zedong (CN, 19, M, ZH) 62. William Herschel (DE, 18, M, DE) 63. Michael Jackson (US, 20, M, EN) 64. Justinian I (MK, 5, M, WR) 65. Augustine of Hippo (DZ, 4, M, AR) 66. Ali (SA, 7, M, AR) 67. Jean-Jacques Rousseau (CH, 18, M, DE) 68. Ernst Haeckel (DE, 19, M, DE) 69. Pliny the Elder (IT, 1, M, IT) 70. Pope Gregory XIII (IT, 16, M, IT) 71. Confucius (CN, -6, M, ZH) 72. Henry VIII of England (UK, 15, M, EN) 73. Thomas Jefferson (US, 18, M, EN) 74. Francisco Franco (ES, 19, M, ES) 75. Georg Wilhelm Friedrich Hegel (DE, 18, M, DE) 76. Pierre Andr Latreille (FR, 18, M, FR) 77. Pope Paul VI (IT, 19, M, IT) 78. Gottfried Wilhelm Leibniz (DE, 17, M, DE) 79. Chiang Kai-shek (CN, 19, M, ZH) 80. John Herschel (UK, 18, M, EN) 81. Elizabeth I of England (UK, 16, F, EN) 82. J. R. R. Tolkien

(ZA, 19, M, WR) 83. Socrates (GR, -5, M, EL) 84. Genghis Khan (MN, 12, M, WR) 85. Qin Shi Huang (CN, -3, M, ZH) 86. Umar (SA, 6, M, AR) 87. Philip II of Spain (ES, 16, M, ES) 88. Frederick the Great (DE, 18, M, DE) 89. Johannes Kepler (DE, 16, M, DE) 90. Emperor Wu of Han (CN, -2, M, ZH) 91. Friedrich Nietzsche (DE, 19, M, DE) 92. Plutarch (GR, 1, M, EL) 93. Thomas Edison (US, 19, M, EN) 94. Max Weber (DE, 19, M, DE) 95. Dante Alighieri (IT, 13, M, IT) 96. Ashoka (IN, -4, M, HI) 97. Tacitus (FR, 1, M, FR) 98. Ernst Mayr (DE, 20, M, DE) 99. Jean-Baptiste Lamarck (FR, 18, M, FR) 100. Elvis Presley (US, 20, M, EN).

Top 100 of global 2DRank names: 1. Adolf Hitler (AT, 19, M, DE) 2. Michael Jackson (US, 20, M, EN) 3. Madonna (entertainer) (US, 20, F, EN) 4. Jesus (PS, -1, M, AR) 5. Ludwig van Beethoven (DE, 18, M, DE) 6. Wolfgang Amadeus Mozart (AT, 18, M, DE) 7. Pope Benedict XVI (DE, 20, M, DE) 8. Alexander the Great (GR, -4, M, EL) 9. Charles Darwin (UK, 19, M, EN) 10. Barack Obama (US, 20, M, EN) 11. Johann Sebastian Bach (DE, 17, M, DE) 12. Napoleon (FR, 18, M, FR) 13. Pope John Paul II (PL, 20, M, PL) 14. Julius Caesar (IT, -1, M, IT) 15. Elizabeth II (UK, 20, F, EN) 16. Albert Einstein (DE, 19, M, DE) 17. Augustus (IT, -1, M, IT) 18. Bob Dylan (US, 20, M, EN) 19. Leonardo da Vinci (IT, 15, M, IT) 20. Mary (mother of Jesus) (IL, -1, F, HE) 21. Charlemagne (BE, 8, M, NL) 22. William Shakespeare (UK, 16, M, EN) 23. Elvis Presley (US, 20, M, EN) 24. Queen Victoria (UK, 19, F, EN) 25. John Lennon (UK, 20, M, EN) 26. George Frideric Handel (DE, 17, M, DE) 27. J. R. R. Tolkien (ZA, 19, M, WR) 28. Muhammad (SA, 6, M, AR) 29. Joseph Stalin (GE, 19, M, WR) 30. Karl Marx (DE, 19, M, DE) 31. Benito Mussolini (IT, 19, M, IT) 32. Franklin D. Roosevelt (US, 19, M, EN) 33. Michael Schumacher (DE, 20, M, DE) 34. Paul McCartney (UK, 20, M, EN) 35. Stephen King (US, 20, M, EN) 36. Henry VIII of England (UK, 15, M, EN) 37. Tokugawa Ieyasu (JP, 16, M, JA) 38. Edgar Allan Poe (US, 19, M, EN) 39. Martin Luther (DE, 15, M, DE) 40. David Bowie (UK, 20, M, EN) 41. Pope Pius XII (IT, 19, M, IT) 42. Alfred Hitchcock (UK, 19, M, EN) 43. Friedrich Nietzsche (DE, 19, M, DE) 44. Vladimir Putin (RU, 20, M, RU) 45. Christopher Columbus (IT, 15, M, IT) 46. Elton John (UK, 20, M, EN) 47. Carl Linnaeus (SE, 18, M, SV) 48. Michelangelo (IT, 15, M, IT) 49. Raphael (IT, 15, M, IT) 50. Roger Federer (CH, 20, M, DE) 51. Cao Cao (CN, 2, M, ZH) 52. Vincent van Gogh (NL, 19, M, NL) 53. Frdric Chopin (PL, 19, M, PL) 54. Steven Spielberg (US, 20, M, EN) 55. Rembrandt (NL, 17, M, NL) 56. Ali (SA, 7, M, AR) 57. Richard Wagner (DE, 19, M, DE) 58. Che Guevara (AR, 20, M, ES) 59. Nelson Mandela (ZA, 20, M, WR) 60. Isaac Asimov (RU, 20, M, RU) 61. Jules Verne (FR, 19, M, FR) 62. Toyotomi Hideyoshi (JP, 16, M, JA) 63. Winston Churchill (UK, 19, M, EN) 64. Paul the Apostle (TR, 1, M, TR) 65. Hirohito (JP, 20, M, JA) 66. 14th Dalai Lama (CN, 20, M, ZH) 67. Franz Liszt (AT, 19, M, DE) 68. Genghis Khan (MN, 12, M, WR) 69. Otto von Bismarck (DE, 19, M, DE) 70. Saint Peter (IL, 1, M, HE) 71. Charlie Chaplin (UK, 19, M, EN) 72. Liu Bei (CN, 2, M, ZH) 73. Oda Nobunaga (JP, 16, M, JA) 74. Suleiman the Magnificent (TR, 15, M, TR) 75. Cyrus the Great (IR, -6, M, FA) 76. George W. Bush (US, 20, M, EN) 77. Agatha Christie (UK, 19, F, EN) 78. Carl Friedrich Gauss (DE, 18, M, DE) 79. Louis XIV of France (FR, 17, M, FR) 80. Saddam Hussein (IQ, 20, M, AR) 81. Pablo Picasso (ES, 19, M, ES) 82. Mariah Carey (US, 20, F, EN) 83. Hans Christian Andersen (DK, 19, M, DA) 84. Plato (GR, -5, M, EL) 85. Britney Spears (US, 20, F, EN) 86. Rafael Nadal (ES, 20, M, ES) 87. George Harrison (UK, 20, M, EN) 88. Margaret Thatcher (UK, 20, F, EN) 89. Jorge Luis Borges (AR, 19, M, ES) 90. Salvador Dal (ES, 20, M, ES) 91. Peter the Great (RU, 17, M, RU) 92. Giuseppe Verdi (IT, 19, M, IT) 93. Sigmund Freud (CZ, 19, M,

WR) 94. Qin Shi Huang (CN, -3, M, ZH) 95. Kangxi Emperor (CN, 17, M, ZH) 96. Martina Navratilova (CZ, 20, F, WR) 97. Charles V, Holy Roman Emperor (BE, 15, M, NL) 98. Zhuge Liang (CN, 2, M, ZH) 99. Constantine the Great (RS, 3, M, WR) 100. Muammar Gaddafi (LY, 20, M, AR)

List of 36 names from the global PageRank list of 1045 names where the birth place in modern geography of countries differs from native language: Jesus (PS AR), Charlemagne (Belgium NL), Immanuel Kant (Russia RU), Moses (Egypt AR), Catherine the Great (Poland PL), Mustafa Kemal Atatürk (Greece EL), Bhumibol Adulyadej (USA EN), Christian V of Denmark (Germany DE), Józef Pilsudski (Litvania WR), Christian IX of Denmark (Germany DE), Philip V of Spain (France FR), Giuseppe Garibaldi (France FR), Muhammad al-Idrisi (Spain ES), Charles XIV John of Sweden (France FR), Leonid Brezhnev (Ukraine WR), George I of Greece (Denmark DA), Juan Carlos I of Spain (Italy IT), Leon Trotsky (Ukraine WR), Golda Meir (Ukraine WR), Valéry Giscard d'Estaing (Germany DE), Magnus IV of Sweden (Noroway WR), Christian I of Denmark (Germany DE), Yitzhak Ben-Zvi (Ukraine WR), Mikhail Bulgakov (Ukraine WR); Kim Jong-il (Russia RU). Lee Myung-bak (Japan JA), Jangsu of Goguryeo (China ZH); Galyani Vadhana (UK EN), Abhisit Vejjajiva (UK EN); Matthias Corvinus (Romania WR), Ferenc Kazinczy (Romania WR), György Kulin (Romania WR), Gabriel Bethlen (Romania WR), Endre Ady (Romania WR), János Arany (Romania WR), Béla Bartók (Romania WR).

List of 53 names from the global 2DRank list of 1616 names where the birth place in modern geography of countries differs from native language: Jesus (PS AR), Charlemagne (BE NL), Isaac Asimov (RU RU), Paul the Apostle (TR TR), Peter Paul Rubens (DE DE), Catherine the Great (PL PL), Julian (emperor) (TR TR), Józef Pilsudski (LT WR), Muhammad Ali of Egypt (GR EL), Juan Carlos I of Spain (IT IT), Shmuel Yosef Agnon (UA WR), Saint Joseph (PS AR), Golda Meir (UA WR), Baibars (UA WR), Levi Eshkol (UA WR), Augustine of Hippo (DZ AR), Yitzhak Ben-Zvi (UA WR), Natan Yonatan (UA WR), Edward Rydz-migy (UA WR), Immanuel Kant (RU RU), Pyotr Stolypin (DE DE), Czeslaw Niemen (BY RU), Moses (EG AR), Albert Camus (DZ AR), Leonid Brezhnev (UA WR), Aharon Barak (LT WR), George Orwell (IN HI), Sergei Korolev (UA WR), Garry Kasparov (AZ TR), Ibn 'Abd al-Barr (ES ES), Georges Simenon (BE NL), Ryszard Kapuściński (BY RU), Mihly Munkácsy (UA WR), Juliusz Slowacki (UA WR), Tadeusz Kościuszko (BY RU), John McCain (PA ES), Maurice, Prince of Orange (DE DE), Zbigniew Herbert (UA WR), Leon Trotsky (UA WR), Charles XIV John of Sweden (FR FR). Lee Myung-bak (JA JA), Jangsu of Goguryeo (CN ZH), Gwanggaeto the Great (CN ZH); Galyani Vadhana (UK EN), Abhisit Vejjajiva (UK EN); Matthias Corvinus (RO WR), Károly Kós (RO WR), László Németh (RO WR), Sándor Körösi Csoma (RO WR), János Bolyai (RO WR), György Kulin (RO WR), Ferenc Kazinczy (RO WR), Béla Bartók (RO WR).

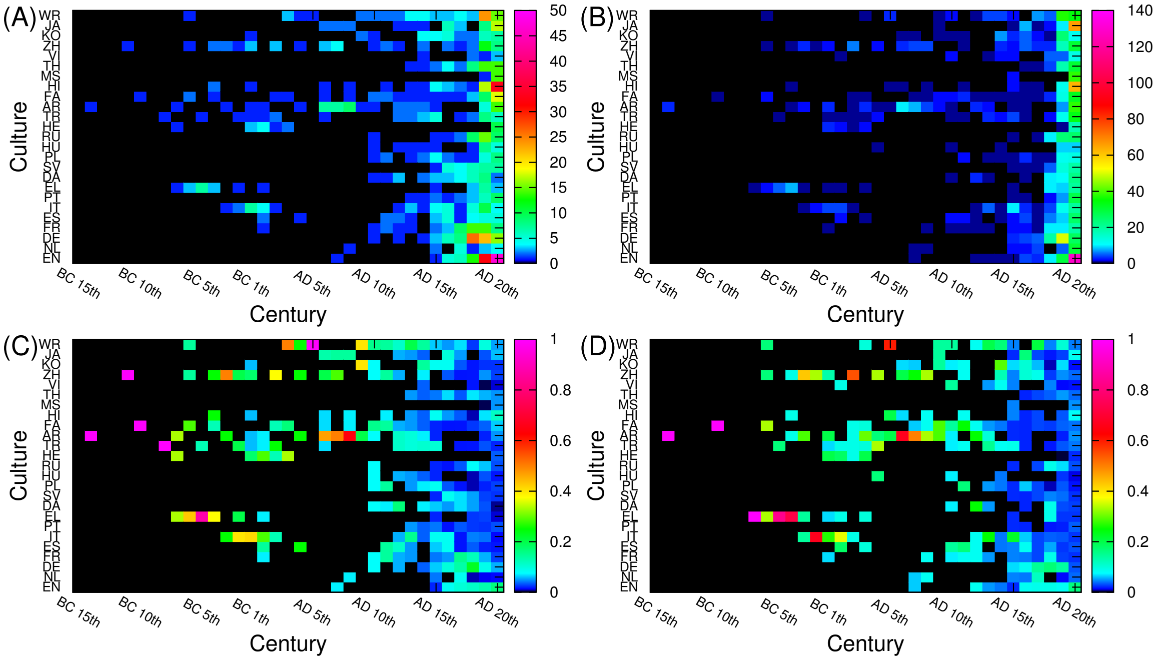

Figure S1. Birth date distribution of historical figures from the global PageRank list (A,C, 1045 persons) and 2DRank list (B,D, 1616 persons). Each historical figure is attributed to her/his own language according to her/his birth place as described in the paper (if the birth place is not among our 24 languages then a person is attributed to the remaining world (WR)). Color in panels (A,B) shows the total number of persons for a given century, while in panels (C,D) color shows a percent for a given century (normalized to unity in each column). This figure give a more distinct separation of cultures (languages) compared to a similar Fig.4 where the distribution over Wikipedia editions is shown on the vertical axis.

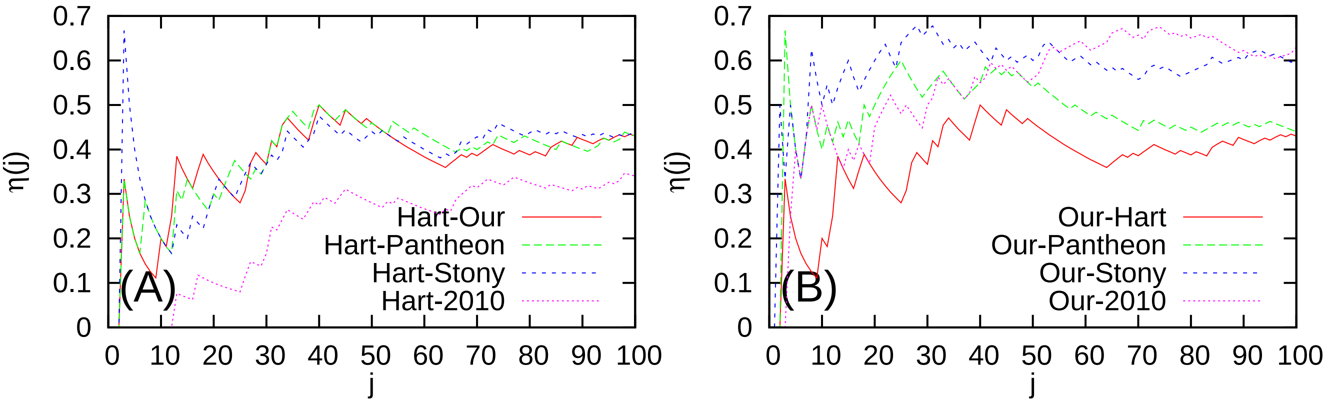

Figure S2. Dependence of fraction η of overlaped persons on rank index of person j. (A) Comparison is done of present study ("our"), PageRank list of English Wikipedia of [15] ("2010"), Stony-Brook list [19], Pantheon MIT project [20] in respect to Hart top 100 list. (B) Same as in (A) but comparison is done in respect to present study.

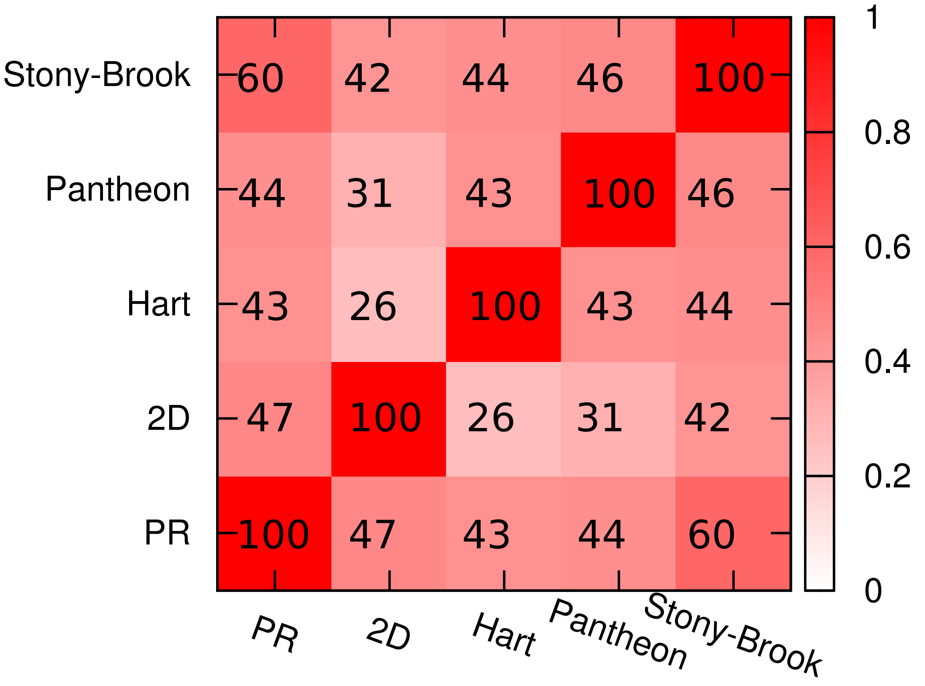

Figure S3. The overlap matrix (in percent) between 5 methods of ranking of top 100 historical figures from lists of Hart, Pantheon, Stony-Brook results and our global PageRank and 2DRank lists; percent or number of persons common for two lists is shown by color and numbers.

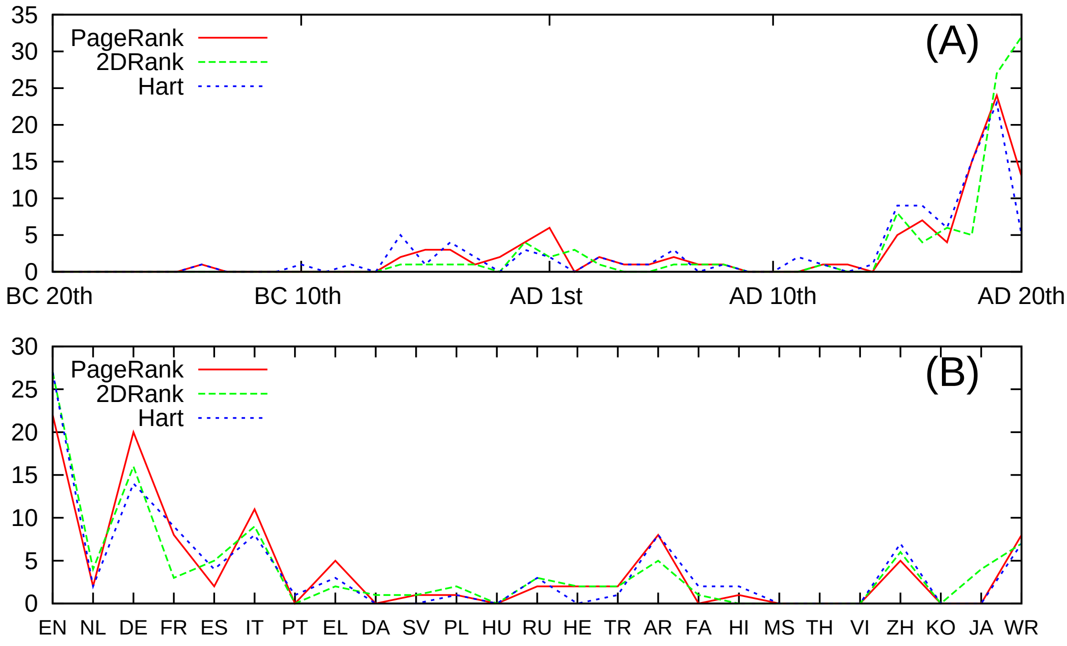

Figure S4. The number of top 100 historical figures, from the list of Hart and our global PageRank and 2DRank lists, are shown as a function of time (for a given century, panel A; one person from Hart's list *Menes*, born in Egypt at BC 32nd and thus attributed to AR, is outside of time range in this panel but he is counted in panel B) and for a given language to which a person is attributed according to her/his birth place (panel B).

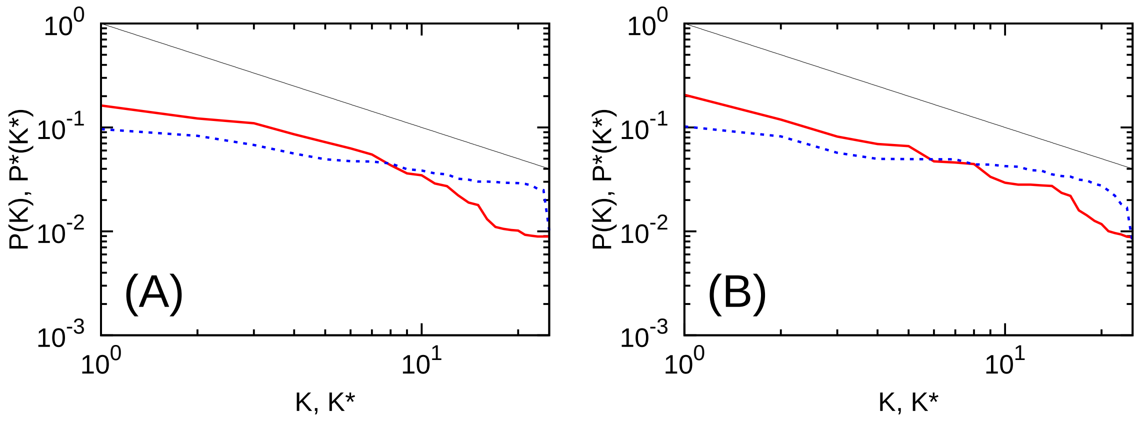

Figure S5. Dependence of probabilities of PageRank P (red) and CheiRank P^* (blue) on corresponding indexes K and K^*. The probabilities are obtained from the network shown in Fig.7 for corresponding panels $(A), (B)$. The straight lines indicate the Zipf's law $P \sim 1/K; P^* \sim 1/K^*$.

Table S1. List of local historical figures for EN category. Here Θ_A is the ranking score of the algorithm A defined in Eq.(1).

	Θ_A	PageRank local figures	Θ_A	2DRank local figures
1	1861	William Shakespeare	1315	Michael Jackson
2	1789	Elizabeth II	991	Madonna (entertainer)
3	1756	Isaac Newton	773	Charles Darwin
4	1173	George W. Bush	754	Barack Obama
5	1101	Barack Obama	664	Elizabeth II
6	932	Charles Darwin	624	Bob Dylan
7	910	Franklin D. Roosevelt	556	William Shakespeare
8	656	George Washington	555	Elvis Presley
9	596	Bill Clinton	550	Queen Victoria
10	564	Winston Churchill	541	John Lennon

Table S2. List of local historical figures for NL category. Here Θ_A is the ranking score of the algorithm A defined in Eq.(1).

	Θ_A	PageRank local figures	Θ_A	2DRank local figures
1	1476	Charlemagne	569	Charlemagne
2	556	Charles V, Holy Roman Emperor	297	Vincent van Gogh
3	83	Maurice Maeterlinck	294	Rembrandt
4	81	William I of the Netherlands	190	Charles V, Holy Roman Emperor
5	78	Beatrix of the Netherlands	138	Beatrix of the Netherlands
6	61	Baruch Spinoza	98	Baruch Spinoza
7	61	Rembrandt	94	Hugo Claus
8	51	Wilhelmina of the Netherlands	91	Johan Cruyff
9	47	Juliana of the Netherlands	76	Louis Couperus
10	39	Christiaan Huygens	75	Pierre Cuypers

Table S3. List of local historical figures for DE category. Here Θ_A is the ranking score of the algorithm A defined in Eq.(1)

	Θ_A	PageRank local figures	Θ_A	2DRank local figures
1	2112	Adolf Hitler	1557	Adolf Hitler
2	1847	Albert Einstein	872	Ludwig van Beethoven
3	1730	Karl Marx	853	Wolfgang Amadeus Mozart
4	996	Wolfgang Amadeus Mozart	840	Pope Benedict XVI
5	925	Martin Luther	733	Johann Sebastian Bach
6	700	Ludwig van Beethoven	651	Albert Einstein
7	610	Johann Sebastian Bach	540	George Frideric Handel
8	570	Johann Wolfgang von Goethe	465	Karl Marx
9	528	Pope Benedict XVI	446	Michael Schumacher
10	417	Richard Wagner	344	Martin Luther

Table S4. List of local historical figures for FR category. Here Θ_A is the ranking score of the algorithm A defined in Eq.(1).

	Θ_A	PageRank local figures	Θ_A	2DRank local figures
1	2208	Napoleon	720	Napoleon
2	1207	Louis XIV of France	268	Jules Verne
3	724	René Descartes	221	Louis XIV of France
4	397	Napoleon III	168	Giuseppe Garibaldi
5	385	Charles de Gaulle	146	Denis Diderot
6	260	Pierre André Latreille	144	Franois Mitterrand
7	167	Tacitus	127	Napoleon III
8	165	Jean-Baptiste Lamarck	121	Nicolas Sarkozy
9	157	Molière	113	Claudius
10	112	Francis I of France	112	Henry IV of France

Table S5. List of local historical figures for ES category. Here Θ_A is the ranking score of the algorithm A defined in Eq.(1).

	Θ_A	PageRank local figures	Θ_A	2DRank local figures
1	276	Francisco Franco	285	Che Guevara
2	195	Philip II of Spain	216	Pablo Picasso
3	119	Pablo Picasso	206	Rafael Nadal
4	82	Lionel Messi	199	Jorge Luis Borges
5	74	Charles III of Spain	198	Salvador Dalí
6	72	Teresa of Ávila	178	Hadrian
7	71	Miguel de Cervantes	105	Shakira
8	70	Ferdinand VII of Spain	100	Francisco Goya
9	66	Alfonso X of Castile	95	Juan Perón
10	65	Ferdinand I, Holy Roman Emperor	94	Augusto Pinochet

Table S6. List of local historical figures for IT category. Here Θ_A is the ranking score of the algorithm A defined in Eq.(1).

	Θ_A	PageRank local figures	Θ_A	2DRank local figures
1	1952	Julius Caesar	689	Julius Caesar
2	1662	Augustus	647	Augustus
3	1476	Christopher Columbus	616	Leonardo da Vinci
4	893	Galileo Galilei	464	Benito Mussolini
5	758	Benito Mussolini	339	Pope Pius XII
6	753	Cicero	330	Christopher Columbus
7	594	Leonardo da Vinci	326	Michelangelo
8	292	Pliny the Elder	322	Raphael
9	288	Pope Gregory XIII	197	Giuseppe Verdi
10	250	Pope Paul VI	172	Galileo Galilei

Table S7. List of local historical figures for PT category. Here Θ_A is the ranking score of the algorithm A defined in Eq.(1).

	Θ_A	PageRank local figures	Θ_A	2DRank local figures
1	91	Getúlio Vargas	109	Ronaldo
2	83	Cristiano Ronaldo	100	Getúlio Vargas
3	74	John VI of Portugal	92	Juscelino Kubitschek
4	71	Luiz Inácio Lula da Silva	91	Rubens Barrichello
5	70	Pedro I of Brazil	90	Joaquim Maria Machado de Assis
6	67	Ferdinand Magellan	89	Fernando Henrique Cardoso
7	66	Maria I of Portugal	82	Luís de Camões
8	64	John I of Portugal	80	José Saramago
9	63	Pedro II of Brazil	79	John VI of Portugal
10	62	Juscelino Kubitschek	77	Oscar Niemeyer

Table S8. List of local historical figures for EL category. Here Θ_A is the ranking score of the algorithm A defined in Eq.(1).

	Θ_A	PageRank local figures	Θ_A	2DRank local figures
1	2237	Aristotle	789	Alexander the Great
2	1949	Plato	207	Plato
3	1771	Alexander the Great	167	Aristotle
4	213	Socrates	108	Pericles
5	178	Plutarch	100	Mustafa Kemal Atatürk
6	153	Mustafa Kemal Atatürk	98	Eleftherios Venizelos
7	123	Sophocles	95	Andreas Papandreou
8	93	Aeschylus	94	Muhammad Ali of Egypt
9	86	Euripides	94	Ioannis Kapodistrias
10	84	Ioannis Kapodistrias	93	Plutarch

Table S9. List of local historical figures for DA category. Here Θ_A is the ranking score of the algorithm A defined in Eq.(1).

	Θ_A	PageRank local figures	Θ_A	2DRank local figures
1	99	Tycho Brahe	210	Hans Christian Andersen
2	94	Ole Rømer	98	Margrethe II of Denmark
3	93	Christian IV of Denmark	95	N. F. S. Grundtvig
4	86	Margrethe II of Denmark	92	Sren Kierkegaard
5	85	Hans Christian Andersen	89	Christian IV of Denmark
6	84	Frederick IV of Denmark	88	Hans Christian Ørsted
7	80	Frederick II of Denmark	86	Anders Fogh Rasmussen
8	78	John Louis Emil Dreyer	84	Carl Nielsen
9	77	Christian VII of Denmark	83	Christian X of Denmark
10	76	Frederick III of Denmark	82	Niels Bohr

Table S10. List of local historical figures for SV category. Here Θ_A is the ranking score of the algorithm A defined in Eq.(1).

	Θ_A	PageRank local figures	Θ_A	2DRank local figures
1	2284	Carl Linnaeus	326	Carl Linnaeus
2	125	August Strindberg	151	Ingmar Bergman
3	98	Alfred Nobel	146	Charles XII of Sweden
4	94	Gustav I of Sweden	116	Astrid Lindgren
5	93	Gustav III of Sweden	100	August Strindberg
6	86	Charles XII of Sweden	98	Carl XVI Gustaf of Sweden
7	82	Gustavus Adolphus of Sweden	92	Evert Taube
8	72	Carl XVI Gustaf of Sweden	89	Jan Myrdal
9	71	Charles XI of Sweden	88	Carl Jonas Love Almqvist
10	67	Charles IX of Sweden	83	Gustav I of Sweden

Table S11. List of local historical figures for PL category. Here Θ_A is the ranking score of the algorithm A defined in Eq.(1).

	Θ_A	PageRank local figures	Θ_A	2DRank local figures
1	864	Pope John Paul II	693	Pope John Paul II
2	94	Catherine the Great	296	Frédéric Chopin
3	88	David Ben-Gurion	135	Catherine the Great
4	80	Casimir III the Great	98	David Ben-Gurion
5	72	Nathan Alterman	95	Bolesaw III Wrymouth
6	69	Lech Walesa	94	Andrzej Wajda
7	66	Lech Kaczyński	93	Nathan Alterman
8	63	Frédéric Chopin	91	Gerhart Hauptmann
9	60	Henryk Sienkiewicz	88	Anton Denikin
10	58	Sigismund I the Old	83	Lech Kaczyński

Table S12. List of local historical figures for HU category. Here Θ_A is the ranking score of the algorithm A defined in Eq.(1).

	Θ_A	PageRank local figures	Θ_A	2DRank local figures
1	93	János Szentágothai	100	Stephen I of Hungary
2	91	Stephen I of Hungary	99	Sándor Petöfi
3	87	Lajos Kossuth	94	Kati Kovács
4	86	Miklós Réthelyi	93	Miklós Horthy
5	80	Béla IV of Hungary	92	Attila József
6	79	Louis I of Hungary	89	Sándor Weöres
7	75	Sándor Petöfi	86	Theodor Herzl
8	67	Miklós Horthy	83	Lajos Kossuth
9	56	Theodor Herzl	81	Miklós Radnóti
10	53	Andrew II of Hungary	77	János Kodolányi

Table S13. List of local historical figures for RU category. Here Θ_A is the ranking score of the algorithm A defined in Eq.(1).

	Θ_A	PageRank local figures	Θ_A	2DRank local figures
1	1123	Immanuel Kant	334	Vladimir Putin
2	1022	Vladimir Lenin	274	Isaac Asimov
3	156	Peter the Great	198	Peter the Great
4	130	Mikhail Gorbachev	171	Vladimir Lenin
5	101	Pyotr Ilyich Tchaikovsky	127	Yuri Gagarin
6	97	Yuri Gagarin	109	Igor Stravinsky
7	97	Alexander Pushkin	100	Menachem Begin
8	91	Vladimir Putin	99	Dmitri Mendeleev
9	89	Nikita Khrushchev	96	Aleksander Griboyedov
10	88	Alexander II of Russia	95	Shimon Peres

Table S14. List of local historical figures for HE category. Here Θ_A is the ranking score of the algorithm A defined in Eq.(1).

	Θ_A	PageRank local figures	Θ_A	2DRank local figures
1	1094	Mary (mother of Jesus)	580	Mary (mother of Jesus)
2	724	Saint Peter	240	Saint Peter
3	138	John the Baptist	171	John the Baptist
4	99	Yitzhak Rabin	99	Saint George
5	95	Yigal Amir	99	Yitzhak Rabin
6	84	Josephus	96	Ariel Sharon
7	81	Tom Segev	92	Benjamin Netanyahu
8	75	Ariel Sharon	85	Ehud Barak
9	65	Benjamin Netanyahu	82	Roni Dalumi
10	54	Herod the Great	79	Moshe Dayan

Table S15. List of local historical figures for TR category. Here Θ_A is the ranking score of the algorithm A defined in Eq.(1).

	Θ_A	PageRank local figures	Θ_A	2DRank local figures
1	973	Paul the Apostle	252	Paul the Apostle
2	925	Herodotus	231	Suleiman the Magnificent
3	133	Strabo	172	Mehmed the Conqueror
4	117	Mehmed the Conqueror	169	Selim I
5	106	Suleiman the Magnificent	142	Abdul Hamid II
6	96	Abdul Hamid II	111	Julian (emperor)
7	93	Pausanias (geographer)	90	Recep Tayyip Erdoğan
8	83	İsmet İnönü	87	Adnan Menderes
9	79	Selim I	85	Lucian
10	79	Hesiod	84	Blent Ecevit

Table S16. List of local historical figures for AR category. Here Θ_A is the ranking score of the algorithm A defined in Eq.(1).

	Θ_A	PageRank local figures	Θ_A	2DRank local figures
1	2282	Jesus	943	Jesus
2	1735	Muhammad	499	Muhammad
3	629	Moses	291	Ali
4	426	Saddam Hussein	219	Saddam Hussein
5	424	Ptolemy	181	Muammar Gaddafi
6	329	Augustine of Hippo	143	Hannibal
7	328	Ali	128	Saladin
8	196	Umar	128	Anwar Sadat
9	147	Anwar Sadat	117	Hosni Mubarak
10	134	Euclid	108	Yasser Arafat

Table S17. List of local historical figures for FA category. Here Θ_A is the ranking score of the algorithm A defined in Eq.(1).

	Θ_A	PageRank local figures	Θ_A	2DRank local figures
1	110	Zoroaster	229	Cyrus the Great
2	101	Darius I	99	Zoroaster
3	100	Mahmoud Ahmadinejad	98	Mohammad Reza Pahlavi
4	97	Mohammad Reza Pahlavi	97	Mohammad Khatami
5	96	Rez Shh	96	Mir-Hossein Mousavi
6	94	Cyrus the Great	95	Ruhollah Khomeini
7	92	Ferdowsi	94	Naser al-Din Shah Qajar
8	90	Ruhollah Khomeini	93	Ali Khamenei
9	89	Naser al-Din Shah Qajar	92	Mohammad Mosaddegh
10	86	Mohammad Khatami	91	Ardashir I

Table S18. List of local historical figures for HI category. Here Θ_A is the ranking score of the algorithm A defined in Eq.(1).

	Θ_A	PageRank local figures	Θ_A	2DRank local figures
1	168	Ashoka	126	Ashoka
2	106	Mahatma Gandhi	108	Akbar
3	100	Benazir Bhutto	99	Indira Gandhi
4	91	Vikramditya	98	Mahadevi Varma
5	90	Shivaji	96	Sanjeev Kumar
6	89	Jawaharlal Nehru	93	Amitabh Bachchan
7	88	Akbar	91	Premchand
8	87	Indira Gandhi	90	Dayananda Saraswati
9	86	Adi Shankara	89	Jaishankar Prasad
10	85	Vishnu Prabhakar	86	Adi Shankara

Table S19. List of local historical figures for MS category. Here Θ_A is the ranking score of the algorithm A defined in Eq.(1).

	Θ_A	PageRank local figures	Θ_A	2DRank local figures
1	96	Mahathir Mohamad	100	Mahathir Mohamad
2	85	Najib Razak	99	Najib Razak
3	84	P. Ramlee	98	Anwar Ibrahim
4	81	Tunku Abdul Rahman	93	Mizan Zainal Abidin of Terengganu
5	79	Abdullah Ahmad Badawi	92	Sudirman Arshad
6	77	Muhyiddin Yassin	91	Tunku Abdul Rahman
7	74	Abdul Razak Hussein	90	Siti Nurhaliza
8	62	Anwar Ibrahim	89	Abdullah Ahmad Badawi
9	58	Hussein Onn	88	Abdul Taib Mahmud
10	37	Mizan Zainal Abidin of Terengganu	84	P. Ramlee

Table S20. List of local historical figures for TH category. Here Θ_A is the ranking score of the algorithm A defined in Eq.(1).

	Θ_A	PageRank local figures	Θ_A	2DRank local figures
1	100	Chulalongkorn	100	Sirindhorn
2	97	Vajiravudh	98	Sirikit
3	96	Mongkut	97	Thaksin Shinawatra
4	94	Buddha Yodfa Chulaloke	94	Taksin
5	92	Nangklao	91	Pridi Banomyong
6	91	Thaksin Shinawatra	90	Yingluck Shinawatra
7	90	Damrong Rajanubhab	88	Srinagarindra
8	89	Taksin	86	Samak Sundaravej
9	88	Plaek Phibunsongkhram	82	Vajiralongkorn
10	87	Prajadhipok	80	Chao Keo Naovarat

Table S21. List of local historical figures for VI category. Here Θ_A is the ranking score of the algorithm A defined in Eq.(1).

	Θ_A	PageRank local figures	Θ_A	2DRank local figures
1	91	Ho Chi Minh	98	Ho Chi Minh
2	71	Ngo Dinh Diem	97	Gia Long
3	62	Minh Mng	96	Minh Mng
4	46	Gia Long	94	Nguyen Hue
5	44	Bo i	86	Le Loi
6	22	Le Loi	84	Tran Hung Dao
7	15	Nhat Linh	83	Vo Nguyen Giap
8	N/A	N/A	82	Tu Duc
9	N/A	N/A	81	Le Thánh Tông
10	N/A	N/A	80	Trung Sisters

Table S22. List of local historical figures for ZH category. Here Θ_A is the ranking score of the algorithm A defined in Eq.(1).

	Θ_A	PageRank local figures	Θ_A	2DRank local figures
1	375	Mao Zedong	306	Cao Cao
2	285	Confucius	243	14th Dalai Lama
3	244	Chiang Kai-shek	234	Liu Bei
4	197	Qin Shi Huang	192	Qin Shi Huang
5	186	Emperor Wu of Han	191	Kangxi Emperor
6	135	Cao Cao	188	Zhuge Liang
7	129	Hongwu Emperor	179	Qianlong Emperor
8	119	Qianlong Emperor	154	Mao Zedong
9	119	Kangxi Emperor	147	Hongwu Emperor
10	94	Sun Yat-sen	146	Sun Yat-sen

Table S23. List of local historical figures for KO category. Here Θ_A is the ranking score of the algorithm A defined in Eq.(1).

	Θ_A	PageRank local figures	Θ_A	2DRank local figures
1	100	Gojong of the Korean Empire	114	Gojong of the Korean Empire
2	98	Kim Il-sung	106	Kim Il-sung
3	95	Sejong the Great	100	Park Chung-hee
4	94	Park Chung-hee	99	Kim Dae-jung
5	93	Taejong of Joseon	97	Roh Moo-hyun
6	92	Syngman Rhee	95	Sejong the Great
7	91	Yeongjo of Joseon	94	Taejo of Goryeo
8	90	Kim Dae-jung	93	Kim Young-sam
9	89	Seonjo of Joseon	92	Jeongjo of Joseon
10	86	Taejo of Joseon	90	Syngman Rhee

Table S24. List of local historical figures for JA category. Here Θ_A is the ranking score of the algorithm A defined in Eq.(1).

	Θ_A	PageRank local figures	Θ_A	2DRank local figures
1	154	Toyotomi Hideyoshi	346	Tokugawa Ieyasu
2	153	Tokugawa Ieyasu	266	Toyotomi Hideyoshi
3	108	Hirohito	252	Hirohito
4	97	Oda Nobunaga	233	Oda Nobunaga
5	86	Emperor Meiji	140	Junichiro Koizumi
6	81	Minamoto no Yoritomo	131	Shinzō Abe
7	76	Junichiro Koizumi	112	Tsunku
8	73	Emperor Tenmu	106	Emperor Meiji
9	70	Natsume Sōseki	100	Koxinga
10	69	Akihito	97	Osamu Tezuka

Table S25. List of local historical figures for WR category. Here Θ_A is the ranking score of the algorithm A defined in Eq.(1).

	Θ_A	PageRank local figures	Θ_A	2DRank local figures
1	1686	Joseph Stalin	529	J. R. R. Tolkien
2	842	Constantine the Great	477	Joseph Stalin
3	564	Gautama Buddha	276	Nelson Mandela
4	506	Sigmund Freud	241	Genghis Khan
5	405	Diocletian	195	Sigmund Freud
6	351	Justinian I	191	Martina Navratilova
7	219	J. R. R. Tolkien	186	Constantine the Great
8	203	Genghis Khan	173	Justinian I
9	138	Avicenna	127	Nikola Tesla
10	129	Rumi	123	Kublai Khan
